# Health seeking behaviour and knowledge on neonatal danger signs among neonatal caregivers in Upper Denkyira East Municipality, Ghana

**DOI:** 10.1186/s12887-023-04430-2

**Published:** 2024-01-08

**Authors:** Philip Gyaase, Edward Aduse-Poku, Mavis Opoku Lanquaye, Emmanuel Boateng Acheampong, David Ben Sampson

**Affiliations:** Nursing and Midwifery Training College, Dunkwa-On-Offin, Ghana

**Keywords:** Health seeking behaviour, Knowledge and neonatal danger signs, Mothers with neonates

## Abstract

**Background:**

The purpose of the project was to assess the health seeking behaviour and knowledge on neonatal danger signs among neonatal caregivers in Upper Denkyira East Municipality.

**Methods:**

The study used a cross-sectional design and simple random sampling technique was employed to sample mothers’ neonates visiting the postnatal clinic in the selected health facilities. The target population was mothers with neonates and above 18 years visiting the health facilities and were willing to be part of the study. Total population for the study was 387 however, 381 responded to the questionnaire. Structured questionnaire was the main data collection tool for the study. Data were analysed with SPSS version 23.0. Logistic regression with Pearson Chi square, *p*-value and odd ratio were the main statistical methods for the data analysis.

**Results:**

The results showed that 138 (36.2%) of the respondents stated that diarrhoea and vomiting constituted the major danger signs that sent their neonates to the hospital. Also the health seeking behaviour of the mothers showed positive results as most of the mothers (77.2%) attended hospital upon seeing neonatal danger sign. Finally, the association between mothers’ socio-demographic characteristics and recognition of neonatal danger signs showed that mothers educational level and occupation were statistically significant (*p*-value = 0.000).

**Conclusion:**

The study concluded that mothers’ knowledge level on neonatal danger signs was high and also caregivers had good health seeking behaviour. It is recommended that community health nurses and midwives should embark on home visits to encourage mothers to practice the knowledge and skills acquired during counselling at the hospital. Mothers should be empowered to make decisions concerning their children’s health care.

**Supplementary Information:**

The online version contains supplementary material available at 10.1186/s12887-023-04430-2.

## Background

One of the most serious public health issues in the world today is neonatal mortality. 75% (75%) of neonatal deaths occur in the first week of neonatal life. Per estimation, four million deaths occur worldwide each year during the first four weeks of neonatal life [1]. 99% of all neonatal deaths take place in low and middle income nations, primarily in sub-Saharan Africa [2]. Most of these neonatal fatalities take place at home, which suggests that either few families are aware of the symptoms of neonatal sickness or that most neonates are not transferred to medical facilities when they are sick [3].

Neonatal danger signs are signs of serious illnesses that affect neonates during their first twenty-eight (28) days of life. Globally, neonatal danger signs are known to be associated with health complications among neonates. These complications include decreased neonatal growth, mental retardation, cerebral palsy among others. The notifiable causes of neonatal danger signs are neonatal jaundice, vomiting, cord sepsis, inability to suck breast milk, convulsions, hyperthermia/hypothermia, etc. [4].

Findings have also indicated that, poor antenatal and post-natal services, poor home care for neonates and decreased nutritional status of mothers contribute to the occurrence of neonatal danger signs [5]. It is also found that, some socio demographic and sociocultural practices which defines health seeking behavior of neonatal mothers has a significant contribution to the occurrence of health related danger signs among neonates [6].

It is known worldwide that, about 7000 neonatal mortalities are recorded every day of which most of these deaths occur in the first twenty-four (24) hours of delivery or within the first twenty-eight (28) days of neonatal birth life. Scientific data have also estimated that, majority of neonatal mortalities are caused by neonatal danger signs [7]. The World Health Organization reported that about four million neonates lose their life during their neonatal period and 98% of these deaths occur in developing countries [7]. The report also estimated that, the risk of developing neonatal danger sign in developing countries is six (6) times higher than in the developed countries [8].

An estimated 1.16 million neonatal deaths occur in Africa each year, with roughly one million of those deaths occurring in the first week of the neonatal period [8]. There is high prevalence of neonatal danger signs in sub-Saharan Africa (31 deaths per 1000 live births) and accounting for 39% of all neonatal deaths worldwide. As a result of this, interventions should be put in place to reduce the prevalence rate of neonatal mortalities [9]. If prompt and appropriate healthcare measures are implemented, the majority of the avoidable causes of neonatal danger signs which account for around two thirds of neonatal diseases might be prevented [10].

A Millennium Development Goal (MDG) was created to lower neonatal morbidities and mortalities. Ghana has acknowledged the tragic effects of neonatal danger signs and has implemented interventions to reduce mortalities [11]. Neonatal mortality has maintained at 59 deaths per 1000 live births despite the nation’s target for infant mortality being 26 deaths per 1000 live births [12]. Improving neonatal health and survival requires timely and appropriate health seeking behavior and timely treatments [13]. This is only possible if mothers have good knowledge and behavior about infant danger signs [14]; [15].

One of the emerging nations in Africa with a high neonatal death rate is Ghana [16]. In Ghana, four out of ten (40%) deaths among children under five are attributable to neonatal mortality [17]. Neonatal mortality increased from 30 to 32 per 1000 live births as of 2019 [4] during the previous five years. Almost half (43%) of all births in Ghana today take place at home [18]. Many of these infants could be subjected to unwholesome care methods, especially if their moms are inexperienced [19]. International organizations have created number of interventions such as immunisation, exclusive breastfeeding and family planning to lower neonatal morbidity and mortality [20].

The Every Newborn Action Plan (ENAP) of the United Nations International Children’s Emergency Fund (UNICEF) and the World Health Organization (WHO) are two examples of such programs [20]. The fundamental goal of this action plan is to address the prevention and management of the major causes of neonatal death [21]. Promoting postnatal care throughout the neonatal period is another area of cooperation between these two organizations [22].

The visits paid by trained health workers to postnatal mothers’ homes, especially during a baby’s first week of life, is one important aspect of the promotion of postnatal care during the neonatal period [23] Another pertinent recommendation made by the WHO is that mothers and their newborns should be released from the hospital after 24 h following an uncomplicated vaginal delivery [24]. However this recommendation also doesn’t seem to be followed [25]. Due to inadequate hospital facilities, many hospitals discharge mothers who delivered earlier than is advised [26]. Because there aren’t enough beds in the maternity hospitals to monitor both laboring women and those in the fourth stage of labor, mothers in the Upper Denkyira East, for instance, who have a normal delivery and their infants appear to be in good health, are released after twelve (12) hours [27].

Numerous of these beneficial suggestions that are intended to address the medical requirements of newborns do not appear to be receiving the required compliance [28]. The Ghana National Newborn Health Strategy and Action Plan, 2018–2022, set a target of lowering neonatal death to 21 per 1000 live births [29]. This is what they hoped to accomplish by endorsing postnatal care. The postpartum period is the perfect time to teach new moms about neonatal care [30]. Global, regional, and local public health concerns surround neonatal morbidity and death [31]. About 38% of neonatal deaths worldwide, primarily in underdeveloped nations, are caused by neonatal danger signals, which are on the rise [32]. The majority of these deaths occur in the first 24 h and the first week of neonatal life, according to the World Health Organization, which estimates that 45% of under-five mortalities occur during the neonatal period [32].

According to estimates, around ten (10) million children die before turning one each year, and 98% of those deaths have place in developing nations [33]. According to reports, there are 130 million newborns born each year, and over 4 million infants die during the first 28 days, with the majority of these deaths occurring in less developed nations [34].

Additionally, it has been discovered that a neonate’s chance of having neonatal danger signs is around six times higher in developing countries than in industrialized ones [35]. The risk of perinatal death is increased by five times during pregnancy and during labor, which has a detrimental effect on the health of the newborn [36]. According to research, the first seven days following delivery account for around 75% of infant fatalities [37].

Neonatal mortality is on the rise, and this rise is perceived as a threat rather than a burden, according to a global agenda on the subject [38]. According to studies, Nigeria has the highest rate of neonatal deaths, and if prompt recognition, diagnosis, and treatment are not provided, a neonate will die within a minute [38]. Another study estimated that about 28% of deaths in children under the age of five are caused by neonatal danger signs.

The majority of neonatal deaths in Ghana are due to neonatal danger signs like infections, birth asphyxia, preterm, and low birth weight [39]. Ghana has a high rate of neonatal danger signs. Due to neonatal danger signs, there has been an increase in hospitalizations and fatalities. Data from the Upper Denkyira East Municipal Health Directorate are already available, and they show that there were 656 admissions overall in 2020, with 41 (6.3%) of those resulting in neonatal fatalities [40]. Between January 2020 and May 2020, there were 264 neonatal admissions to hospitals, out of which 18 (6.8%) fatalities were noted [40].

Although studies have demonstrated that neonates can display dangerous neonatal symptoms, other researchers believe that this issue is more prevalent in the study site [41]. Postpartum mothers’ awareness of neonatal care procedures is improved by health education [42]. If moms are knowledgeable on how to provide good infant care, these neonatal deaths may be decreased [41]. Some women lack knowledge and expertise about newborn routines and care.

Some adolescent moms exhibit poor newborn care, as evidenced by their delayed breastfeeding beginning and incorrect feeding habits [43].

Any action or inaction made by mothers who believe their newborns exhibit neonatal danger signals or are ill with the intention of finding a suitable solution is considered to be a mother’s health seeking behavior [41] After birth, the health of the neonate largely depends upon the care and practices adopted by their mothers; hence, the need to assess health seeking behaviour, knowledge and association between the socio-demographic characteristics of mothers and the knowledge on neonatal danger signs on neonatal danger signs among mothers in the Upper Denkyira East Municipality to provide information to educate and empower mothers to reduce morbidity and mortality during neonatal period.

## Methods

### The study area

The study took place in the Upper Denkyira East Municipality (UDEM) of Ghana. This municipality is one of the twenty-two (22) Administrative Metropolitan, Municipalities and Districts of the Central Region of Ghana. The Administrative Capital is Dunkwa-On-Offin. The upper Denkyira East Municipality covers a total land area of 1,020 square kilometres, which is about 10% of the total land area of the Central Region [44].

The population of Upper Denkyira East Municipality according to the 2021 Population and Housing Census is 110,141 representing 3.3% of the region’s total population. About 50% (50%) of the communities in municipality are rural without hospitals. They access health care from under resourced clinics and Community-based Health Planning and Services (CHPS) compounds. The study Area has twenty three health institutions made up of hospitals, health centers, rural clinics, and private clinics. Others are private maternity homes and Community Health Planning and Services (CHPS) Compounds. The study sites contained eight (8) health facilities in the municipality as shown in Table 1. The health facilities have an Out-patient Department (OPD), an Antenatal Clinic (ANC), Obstetric and Gynaecological unit, post-natal unit, Neonatal Intensive Care unit, wards and functional Medical Laboratory Unit.

### Study design

The study employed a cross-sectional study designs. A cross-sectional design deals with situations that occur, performance that prevail and beliefs that are on-going and styles that are emerging [45]. The cross-sectional survey design method uses questions to answer issues of concern to the researcher. The design was chosen due to its ability to take data about people for a specific period of time without embarking on any follow-up. A cross sectional design gave the researchers the opportunity to examine the health seeking behaviours and knowledge of lactating mothers in their natural environment. The features of the population were also described as well as their responses. The quantitative approach was adopted for the study. This approach allowed the researchers to use a representative sample size to generalise for the entire study population. With this approach, observable data were gathered to answer the research questions using statistical, computational or mathematical techniques.

### Study population

The target population was mothers with neonates and above 18 years visiting the health facilities and were willing to be part of the study. Fathers and other surrogate caregivers were excluded.

### Sample size and sampling technique

A simple random sampling technique was employed in the sampling of the study participants. A random sampling of mothers with neonates and were 18 years and above were chosen from eight (8) health facilities in the Upper Denkyira East Municipality. Proportionate stratified sampling method was used to select the actual respondents depending on the population size of mothers with neonates at the health facilities during the post-natal clinic. This method gave a fair representation of the study population from each health facility after the sample size had been calculated. The researcher reviewed the daily clinic attendance to determine the list of eligible participants. Using a flip of the fifty (50) Ghana pesewas coin, mothers who met the eligibility criteria were selected. Mothers whose coin showed the Ghana Coat of Arms side were selected. Data were collected over a period of three weeks.

The data collection started on 2nd November to 23rd November, 2022. The study employed Cochran Formula to determine the study minimum sample size; $n = Z\Lambda 2\left( {{\rm{pq}}} \right) \div (e\Lambda {2^2})$. Where n was the minimum sample population thus proportion of population that had the variable of interest, (p=0.40) q was the proportion of the population without the actual variable of interest, (q=1-p) Z was the confidence level set at 95%, e’ was the sampling error or level of precision set at 5% using the above formulae. With 5% unresponsive rate, the required sample size for the study was 381. This was extrapolated from the total mothers of 2650 from January to October, 2022. Stratified sampling technique was used to select respondents from each facility. This was done by using a formula A/B*C as shown in Table 1 below. For example sample size for Dunkwa Gov’t Hospital was 415/2650*381 = 60. Same procedure was used for the other health facilities.

**Table 1:** Proportionate stratified sampling of respondents

Facility (A)	Population of mothers	Number sampled
Dunkwa Gov’t Hospital	415	60
Kyekyewere Health Center	323	47
Asikuma Health Center	294	42
Oponsu Health Center	237	34
Mrayeim Health Center	216	31
St Mark Hospital	383	55
Pentecost Hospital	379	54
Redeemer Hospital	403	58
Total	2650 (B)	381 (C)

### Study variables

The study variables were divided into two, the dependent and the independent variables. The dependent variables were variables that were tested and measured in the research work whilst the independent variables were the variables that were controlled in the research work to test the effects on the dependent variables as shown in Table 2. The table shows the logical framework of the various variables assessed with their operational definitions, level of measurement and type of variable.

**Table 2:** Logical Framework of the study variables

Variable	Operational Definition	Level of Measurement	Type of Variable
Age	Above eighteen years	Ratio	Continuous
Marital status	Married, Single, Co-habiting	Nominal	Categorical
Education level	No Formal Education, Formal Education.	Nominal	Categorical
Employment	Employed, Unemployed	Nominal	Categorical
Umbilical Discharge	Presence of wet and smelly umbilical cord.	Nominal	Categorical
Yellowing of neonatal body	Yellowish of neonatal skin and face	Nominal	Categorical
Temperature	Neonatal body too cold and hot	Ratio	Continuous
Poor Feeding	Neonatal inability to adequately suck breast milk	Nominal	Categorical
Convulsion	Continuous involuntary movement of the body	Nominal	Categorical
Vomiting	Excess fluid from neonatal mouth	Nominal	Categorical
Cultural practices, beliefs	Practices that prevent mothers to seek healthcare for sick neonates.	Nominal	Categorical
Accessibility to healthcare	Closeness of health facility for medical care	Nominal	Categorical
Altitude of health staff	Health workers behaviour that prevent mothers to seek health for their neonates when sick.	Nominal	Categorical
Decision making with regards to neonatal health	Power to make decision when baby is sick and when to take to hospital	Nominal	Categorical

### Data Collection instrument

Data collection was done by the use of a structured questionnaire. The questionnaire comprised four (4) sections which are: personal and general information on respondents, knowledge of mothers on neonatal danger signs, health seeking behaviours of mothers on neonatal danger signs and Socio-demographic characteristics of mothers that were associated with neonatal danger signs. The principal investigator and the research assistants helped the mothers who could not understand certain concepts during the data collection to prevent misinterpretations. The researchers used three (3) weeks for the data collection since some of the health facilities were dispersed widely across the municipality.

### Pre-testing

In the Diaso Hospital in the Upper Denkyira West District, pre-testing of the data collection instruments took place. The pre-test facility was situated outside the study area, but in terms of personnel, facilities given to clients and the configuration of the wards, it had similar characteristics. The pre-testing helped classify certain difficulties that were linked to the understanding of the respondents. The researcher pre-tested the questionnaire on 30 mothers with neonates to check for reliability and validity of the instrument. Corrections in the questionnaire were made by experts and the research team members. The Cronbach Alpha co-efficient was calculated for the questionnaire and yielded 0.803 hence reliable and valid study instrument.

### Data Analysis

The data for the study were analyzed using SPSS version 23.0. The categorical variables analysed using descriptive statistics and data presented in percentages, mean, standard deviation. The analysis of association between normally distributed variable was done using Pearson Chi-square and *p*-value data analysis tools were used to assess the association between the respondents’ sociodemographic characteristics and knowledge on neonatal danger signs. A probability value of less than 0.05 was considered as having a significant statistical association. Missing data were addressed by referring to the primary data to enter any oversight data. Any data genuinely missing from the primary data were excluded. Strobe checklist was used in reporting this cross-sectional study. To reduce missing data, the investigator made sure respondents completed all items on the questionnaire before retrieval.

### Ethical consideration

Ethical approval of the study was obtained from the Ghana Health Service Ethics Review Committee with identification number: GHS-ERC 037/10/22. Data Collection commenced after ethical approval was granted. Permission was also sought from the Municipal Director of Health Services and the Management of the various Health Facilities used for the study. The purpose of the study was discussed with the respondents for their informed consent. Written informed consent was signed by the respondents prior to participating in the study. Since the study used some illiterates, an informed consent was obtained from their legal guardians. Each participant was taking through the study purpose and eligible persons were made to sign a consent form to indicate their acceptance to be part of the study. Respondents who could not read or write were given interpreters to translate the English to their local language (Twi). Respondents were assured of confidentiality and anonymity for the information provided.

## Results

### Socio-demographic characteristics of the respondents

Most of the respondents 207(54.3%) were within the age range of 21–30 while 63(16.5%) were below 21 years. Majority of the respondents 247(64.8%) were Christians as against 134(35.2%) who were Moslems. An overwhelming majority of the respondents 260(68.2%) were Akans with 33 (8.7%) been Ga-Adangbe. Again, 138(36.2%) of the respondents had Tertiary educational certificate followed by Senior High 96(25.2%) while minority 30(7.9%) had no formal education. Public workers formed 145(38.1%) of the respondents while 46(12.1%) were farmers. Lastly, an overwhelming majority of the respondents 347(91.1%) delivered at the hospital while 34(8.9%) delivered at home as shown in Table 3.

**Table 3:** Socio-demographic Characteristics of the Respondents

Variables	Frequency n = 381	Percent
**Age range**		
Below 21 years 21–30 years 31–40 years Mean = 27; SD 15.5	63 207 111	16.5 54.3 29.1
**Religion**		
Christianity Moslem	247 134	64.8 35.2
**Tribe**		
Akan Ewe Ga-Adangbe Northerner	260 48 33 40	68.2 12.6 8.7 10.5
**Educational level**		
No formal education Primary JHS SHS Tertiary	30 31 86 96 138	7.9 8.1 22.6 25.2 36.2
**Occupation**		
Trader Artisan Farmer Public worker Unemployed	58 75 46 145 57	15.2 19.7 12.1 38.1 15.0
**Place of delivery**		
Hospital Home	347 34	91.1 8.9

### Knowledge of mothers on neonatal Danger signs

Table 4 shows the knowledge of mothers on neonatal danger signs; majority of the respondents 244(64.0%) had heard about neonatal danger signs before. Out of the 244 respondents who had heard about neonatal dangers, 160 (65.5%) had their information from health workers as against 19 (7.7%) who had it from friends. Majority of the respondents 201 (82.3%) defined neonatal danger signs as conditions that affect neonates with serious outcomes whilst 12 (4.9%) had no idea. Also, most of the respondents 187 (76.6%) recognized unhealthy looks of their babies as danger sign whereas 20 (8.1%) said when babies had fever. Majority of the respondents 231 (94.6%) took their babies to the hospital whilst 13 (5.3%) gave babies paracetamol.

**Table 4:** Knowledge of Mothers on Neonatal Danger Signs

Variables	F = 381	Percent (%)
**Have you heard about neonatal danger signs before?**		
Yes No	244 137	64.0 36.0
**If yes, where did you hear it from (n = 244)**		
Health workers Friends Media	160 19 65	65.5 7.7 26.6
**What are neonatal danger signs?**		
Conditions that affect neonates that may lead to serious outcomes Conditions that affect only neonates Don’t know	201 31 12	82.3 12.7 4.9
**How will you recognize neonatal danger signs?**		
When baby looks unhealthy When baby is crying When baby has fever Baby has diarrhoea	187 13 20 24	76.6 5.3 8.1 9.8
**What do you do when your baby has danger signs**		
Give paracetamol Go to hospital	13 231	5.3 94.6

### Conditions that would make mother send her neonate immediately to a health facility

**Fig. 1:**
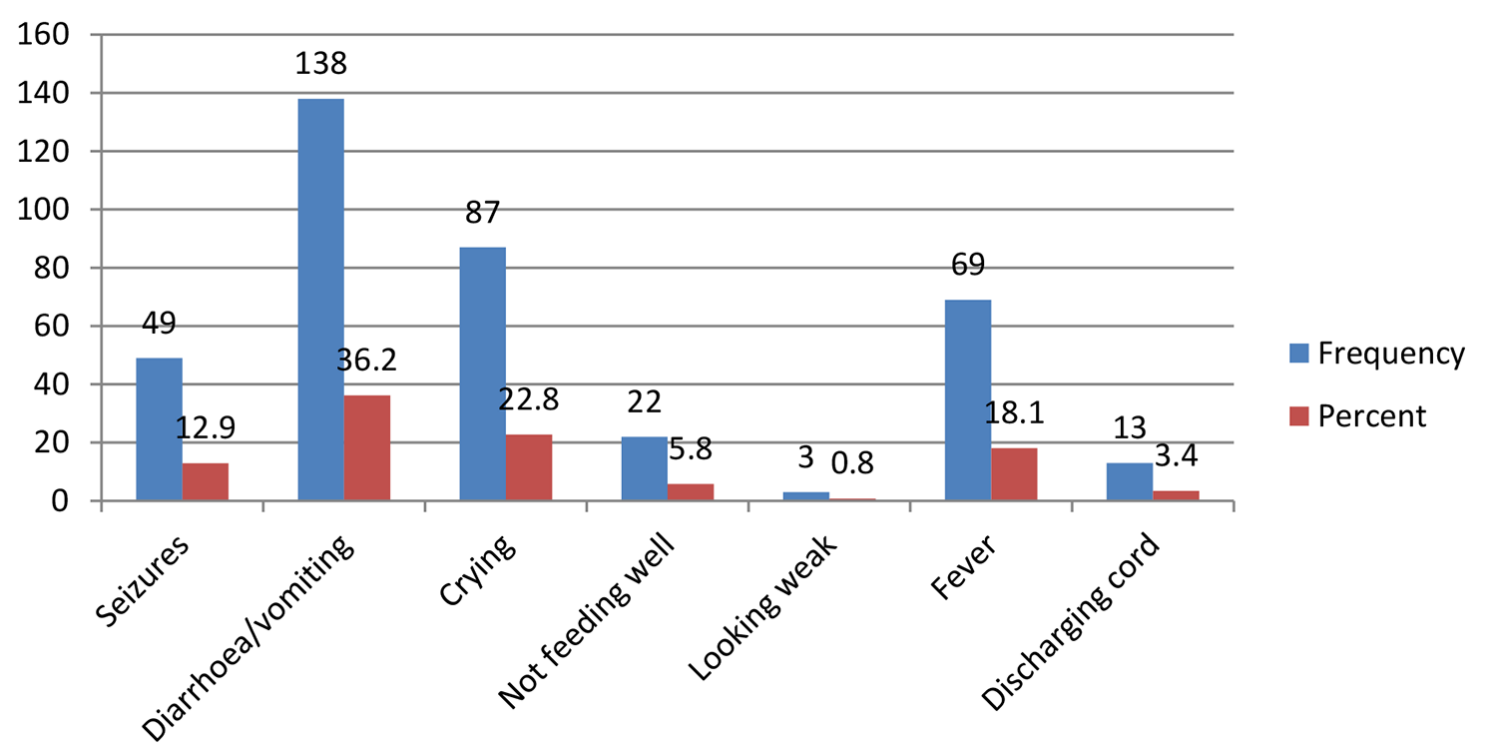
Conditions that would make mother send her neonate immediately to a health facility

From Figs. 1 and 138(36.2%) of the respondents stated that diarrhoea/vomiting constituted the major danger sign that sent their neonates in to health care facilities followed by crying 87(22.8%) whilst the few of them 3(0.8%) said when the neonates were looking weak in appearance.

### Health seeking behaviours of mothers with neonatal danger signs

Table 5 depicts the health seeking behaviours of the respondents; most of the respondents 360(94.5%) sought health care at the hospital whilst 21 (5.5%) used the chemical shops. Also, almost half of the respondents 178(49.4%) received treatment from the public hospital as against 72 (20.0%) respondents who used the private hospitals. Again, more than half of the respondents 203 (56.3%) had visited the hospital twice as against 12 (3.3%) who had visited four times with their current babies. Furthermore, majority of the respondents 274(76.1%) gave their babies paracetamol before taken them to the hospital whilst 7 (1.9%) bathed babies with cold water. Again, most of the respondents 250 (69.4%) prescribed the above treatment themselves whilst 40 (11.1%) had the prescription from friends.

**Table 5:** Health seeking behaviours of mothers with neonatal danger signs

Variables	Frequency	Percent (%)
**Where do you seek for health when your baby is sick**		
Hospital Chemical shop Total	360 21 381	94.5 5.5 100.0
**If hospital, where do you receive care?**		
Public hospital Mission hospital Private hospital Total	178 110 72 360	49.4 30.5 20.0 100.0
**How many times have you visited a hospital with this baby**		
Once Twice Thrice Four Total	45 203 100 12 360	12.5 56.3 27.7 3.3 100.0
**What do you do before taking your baby to the hospital?**		
Give paracetamol Give ORS Bath baby with cold water Total	274 79 7 360	76.1 21.9 1.9 100.0
**Who prescribed the treatment above?**		
Self Husband Friends Total	250 70 40 360	69.4 19.4 11.1 100.0

### Who normally advised mothers to go to the hospital?

**Fig. 2:**
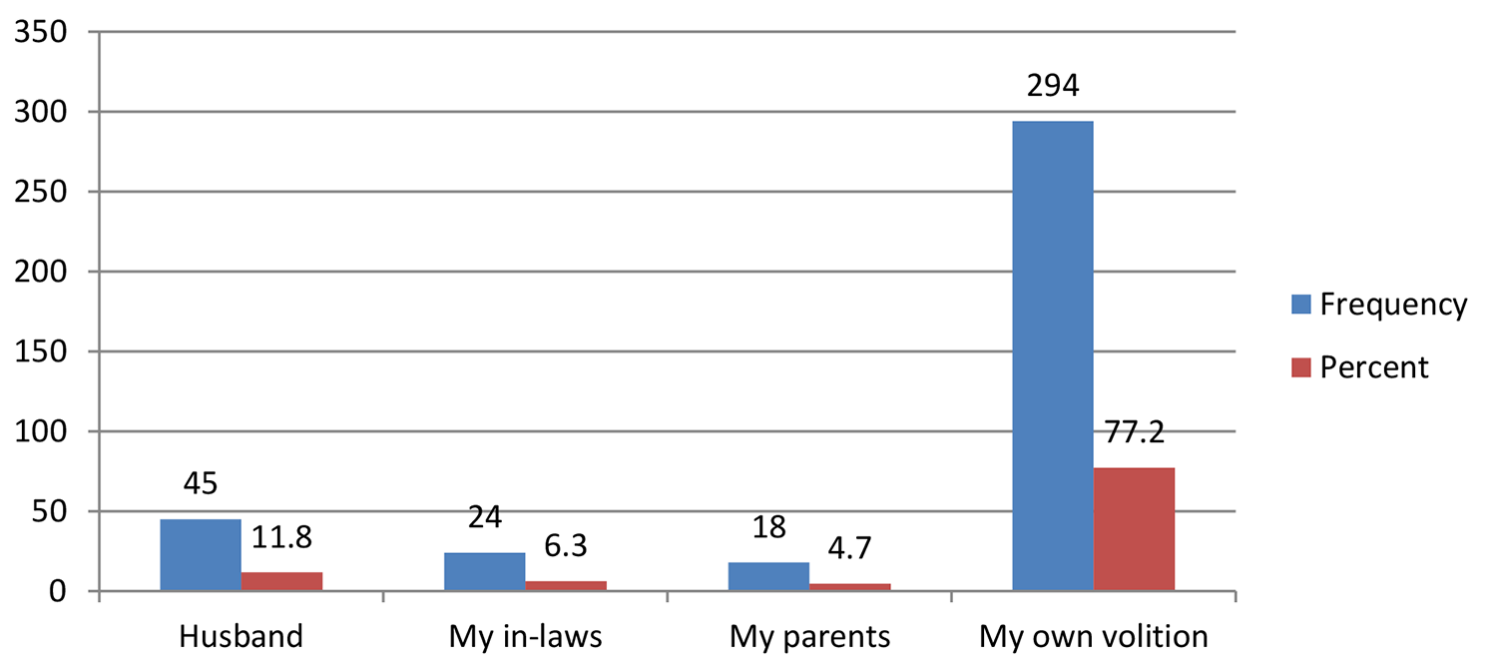
Who Normally Advised Respondents to go to the Hospital

Figure 2 below indicates those who advised respondents to go to the hospital; an overwhelming majority of the respondents 294(77.2%) indicated that they went to the hospital on their own volition whilst 18(4.7%) went to the hospital upon the advice of their parents.

### The Association between the Socio-demographic characteristics of mothers and the knowledge on neonatal danger signs

Table 6 shows the bivariate analysis of the association between the socio-demographic characteristics of mothers and their knowledge on neonatal danger signs, the following variables proved statistically significant; age (*p*-value = 0.005), marital status (*p*-value = 0.000), religion (*p*-value = 0.004), tribe (*p*-value = 0.000), educational level (*p*-value = 0.000) and occupation (*p*-value = 0.000) whereas place of birth was not statistically significant with *p*-value = 0.165.

**Table 6:** The bivariate analysis of the association between the socio-demographic characteristics of mothers and the knowledge on neonatal danger signs

Variables	Knowledge on conditions that neonates would be sent to the hospital							*χ2(p-value)*
	Seizures f(%)	Diarrhoea/Vomiting f(%)	Crying f(%)	Not feeding well f(%)	Looking weak f(%)	Fever f(%)	Discharging cord f(%)	
**Age range**								
Below 21years 21-30years 31-40yreas Total	6(1.6) 25(6.6) 18(4.7) 49(12.9)	15(3.9) 76(19.9) 47(12.3) 138(36.2)	21(5.5) 45(11.8) 21(5.5) 87(22.8)	3(0.8) 9(2.4) 10(2.6) 22(5.8)	0(0.0) 3(0.8) 0(0.0) 3(0.8)	18(4.7) 39(10.2) 12(3.1) 69(18.1)	0(0.00) 10(2.6) 3(0.8) 13(3.4)	28.278*(0.005)*
**Marital status**								
Single Married Co-habiting Divorced Total	6(1.6) 37(9.7) 6(1.6) 0(0.00) 49(12.9)	30(7.9) 69(18.1) 39(10.2) 0(0.00) 138(36.2)	36(9.4) 36(9.4) 15(3.9) 0(0.00) 87(22.8)	3(0.8) 16(4.2) 3(0.8) 0(0.0) 22(5.8)	3(0.8) 0(0.0) 0(0.0) 0(0.0) 3(0.8)	21(5.5) 36(9.4) 9(2.4) 3(0.8) 69(18.1)	7(1.8) 6(1.6) 0(0.0) 0(0.0) 13(3.4)	56.906*(0.000)*
**Religion**								
Christianity Moslem Total	33(8.7) 16(4.2) 49(12.9)	93(24.4) 45(11.8) 138(36.2)	48(12.6) 39(10.2) 87(22.8)	16(4.2) 6(1.6) 22(5.8)	3(0.8) 0(0.0) 3(0.8)	51(13.4) 18(4.7) 69(18.1)	3(0.8) 10(2.6) 13(3.4)	19.280*(0.004)*
**Tribe**								
Akan Ewe Ga-Adangbe Northerner Total	34(8.9) 12(3.1) 3(0.8) 0(0.0) 49(12.9)	95(24.9) 15(3.9) 18(4.7) 10(2.6) 138(36.2)	57(15.0) 18(4.7) 3(0.8) 9(2.4) 87(22.8)	10(2.6) 0(0.0) 6(1.6) 6(1.6) 22(5.8)	3(0.8) 0(0.0) 0(0.0) 0(0.0) 3(0.8)	54(14.2) 0(0.0) 3(0.8) 12(3.1) 69(18.1)	7(1.8) 3(0.8) 0(0.0) 3(0.8) 13(3.4)	74.182*(0.000)*
**Educational level**								
No formal edu. Primary JHS SHS Tertiary Total	3(0.8) 3(0.8) 3(0.8) 9(2.4) 31(8.1) 49(12.9)	15(3.9) 6(1.6) 31(8.1) 27(7.1) 59(15.5) 138(36.2)	0(0.0) 9(2.4) 21(5.5) 24(6.3) 33(8.7) 87(22.8)	0(0.0) 0(0.0) 4(1.0) 12(3.1) 6(1.6) 22(5.8)	0(0.0) 3(0.8) 0(0.0) 0(0.0) 0(0.0) 3(0.8)	9(2.4) 6(1.6) 24(6.3) 21(5.5) 9(2.4) 69(18.1)	3(0.8) 4(1.0) 3(0.8) 3(0.8) 0(0.0) 13(3.4)	105.475*(0.000)*
**Occupation**								
Trader Artisan Farmer Public worker Unemployed Total	0(0.0) 6(1.6) 6(1.6) 28(7.3) 9(2.4) 49(12.9)	21(5.5) 24(6.3) 21(5.5) 63(16.5) 9(2.4) 138(36.2)	9(2.4) 15(3.9) 6(1.6) 36(9.4) 21(5.5) 87(22.8)	0(0.0) 9(2.4) 4(1.0) 9(2.4) 0(0.0) 22(5.8)	3(0.8) 0(0.0) 0(0.0) 0(0.0) 0(0.0) 3(0.8)	18(4.7) 21(5.5) 3(0.8) 9(2.4) 18(4.7) 69(18.1)	7(1.8) 0(0.0) 6(1.6) 0(0.0) 0(0.0) 13(3.4)	136.249*(0.000)*
**Place of delivery**								
Hospital Home Total	46(12.1) 3(0.8) 49(12.9)	125(32.8) 13(3.4) 138(36.2)	78(20.5) 9(2.4) 87(22.8)	22(5.8) 0(0.0) 22(5.8)	3(0.8) 0(0.0) 3(0.8)	60(15.7) 9(2.4) 69(18.1)	13(3.4) 0(0.0) 13(3.4)	9.156*(0.165)*

## Discussions

### Knowledge of mothers on neonatal danger signs

The study revealed among other findings that most mothers had good knowledge on neonatal danger signs that call for immediate care at health facility. This suggests that mothers could recognize the warning signs of newborn illnesses and took their children to the hospital. This finding contradicts a study conducted in rural Northern Ghana which indicated only 20% of mothers could name at least four newborn danger signs [5]. The reason for the difference might be due to the fact that the studies were conducted in different regions and municipalities where the respondents had different characteristics. Also, the period for conducting both studies could account for the differences. Additionally, the knowledge finding in this study was similar to the finding found in a study conducted in Ethiopia, where it was discovered that 84% of mothers had enough knowledge on neonatal danger signs [8].

Specifically, on source of mothers information, more than half (64.0%) of the mothers heard about neonatal danger signs from the health workers. Majority of the respondents (82.3%) defined neonatal danger signs as conditions that affect neonates with serious outcomes whilst few of them had no idea. Also, most of the respondents (76.6%) recognized unhealthy looks of their babies as danger sign whilst other said when babies had fever. Majority of the respondents took their babies to the hospital whilst few of them gave babies paracetamol. This is consistent with Word Health Organization’s 2018 recommendation on care of the newborns [3]. This might suggest that most mothers are adhering to the WHO recommendations on how to care for babies to reduce neonatal mortality. Complications and neonatal fatalities may be decreased if postnatal mothers are able to quickly recognize danger signs and take appropriate measures.

The knowledge of mothers on neonatal danger signs corroborates with several studies including [9, 12 & 16] where mothers were able to list about five neonatal danger signs. Also, most of the mothers agreed with the previous researchers that they could detect some neonatal danger signs including fever, inability to feed, weakness, convulsions, coldness, yellowing of the skin, difficulty breathing, boils and/or rashes, and rapid breathing [12].

In this current study mothers were able to identify the following neonatal danger signs: diarrhoea/vomiting, crying, fever, convulsions, not feeding properly, discharging cord, and weak appearance of the neonates. Spitting up and vomiting may typically be distinguished by seasoned mothers, but first-time parents may need to consult a doctor or nurse.

In a related study involving 420 mothers who had a live birth at home within the previous 12 months and their infants aged 12 months or less, a similar trend was seen in a rural location in the Northern Region of Ghana [27]. The study discovered that only 2.5% of the mothers were unaware of any danger signs in the newborn, 77.2% of respondents were aware of one to three newborn danger signs [27]. Assuming all other factors remain constant, it is anticipated that teaching mothers who visit postnatal clinics will improve mothers’ habits about newborn care. However, a study observed alarmingly high rates of newborn care practices among mothers who had reportedly received cord care instruction from medical professionals. They discovered certain gaps in knowledge on newborn care procedures in cities, which are frequently multi-cultural and benefit from better access to healthcare [26].

### The health seeking behaviour of mothers with neonates

This objective sought to assess mothers’ health seeking behaviour regarding their neonates with danger signs. The results revealed that most of the mothers attended postnatal clinic. Again, most of them attended the public hospitals and this might be due to the fact that the majority of the hospitals in the municipality are public (belong to the government). These findings support similar a study in India Uttar Pradesh which stated that mothers who attended antenatal and postnatal clinics were able to determine neonatal danger signs than mothers who used homebased treatment [30]. Hospital attendance by mothers at delivery was significantly associated with neonatal care practice when compared to mothers who did not attend hospital during and after delivery [30]. This implies that hospital attendance is crucial in the determination and care of the neonates. In a related study, it was discovered that mothers who visited a clinic after giving birth and who received advice on how to care for the neonate were more likely to attend hospitals upon seeing danger signs than mothers who did not visit a health facility and who did not receive neonatal care advice [15].

The results of the current study showed that the majority of the mothers had attended hospitals twice after delivery for treatment. This finding supports a study that found mothers who attended hospitals more than once after delivery were more likely to recognise danger signs of their babies and seek for high-quality neonatal care at the hospitals [24]. This might suggests that health workers educate mothers during their visit to the health facilities for neonatal care. This education helps the mothers to be abreast with neonatal danger signs and how to deal with them. Similar findings were seen in a study in Ethiopia, which indicated that postnatal counselling received and place of care after delivery affected mothers health seeking behaviours for their babies [12].

Regular hospital attendance made the mothers more knowledgeable on the danger signs of the neonates. This result is consistent with a study done in India, where it was discovered that a mother’s hospital attendance had a substantial relationship to safe cord care and the identification of other neonatal risk signs. The same study discovered that women with one hospital attendance had lower rates of recommended neonatal care practices than mothers with several attendance [33].

Again, it was discovered that most of the mothers gave paracetamol to their babies before taking them to the hospital. This shows that mothers always try their own remedies before going to the hospital in situation where there is no improvement in the disease conditions. This behaviour of mothers could be due to the time that they spend whenever they visit the health facility for care. More than 50% of neonatal deaths are a result of health personnel attitudes toward mothers and their babies, according to demographic and health survey data from 40 countries gathered between 2015 and 2020 [7]. Numerous nations have incorporated various methods into their health systems to make it easier to identify these health issues and lower infant mortality [7]. The WHO’s Integrated Management of Newborn and Childhood Illness (IMNCI) program, which was designed, focuses on identifying general neonatal danger signs in newborns who present with illness at medical facilities [16].

The incidence of under-five mortality has steadily declined worldwide during the past few decades. Nearly two-thirds of all deaths in the first year of life and 40% of deaths before the age of five occur during the neonatal era [14]. Due to a shift in certain healthcare professionals’ attitudes about sick babies and their mothers, this achievement has been curtailed. There is evidence that reducing the percentage of home and self-treatment could reduce neonatal deaths [4].

In terms of prescription of treatment for babies, the majority of respondents did so by themselves. Understanding the patterns and factors that influence how mothers and families seek care for their babies is essential to designing effective measures to increase infant survival. One of the key methods to lower infant mortality in underdeveloped countries is to improve mothers’ health seeking behaviors [18]. According to the WHO, obtaining quick and proper care might minimize the mortality rate of children with acute respiratory infections by 20%. Early detection of neonatal danger signs and the provision of high-quality curative healthcare treatments for sick neonate are significant strategies to lower neonatal mortality [2].

The likelihood that an infant would survive could be considerably increased by early diagnosis of neonatal morbidity. However, the mother, father, grandparents, and other close relatives may have an impact on a mothers’ early interest in health. In Africa, grandparents and other family members play a significant role as gatekeepers for child care. Their activities can occasionally prevent children from obtaining medical attention [19]. This finding is contrary to the current study’s findings, where most women visited the hospital on their own free will. WHO reports that due to poor health seeking behavior, children have the highest risk of dying in the first 28 days of life, with an average global rate of 18 fatalities per 1000 live births in 2018.

One of the most frequent causes of neonatal mortality in underdeveloped countries is health seeking behavior. Some people believe that the current efforts to lower neonatal mortality are hampered by an inadequate understanding of the social and cultural factors that influence health, as well as the danger signs of the neonate and the implementation of effective ways to lessen their effects. At home, where few women seek medical attention for symptoms of neonatal sickness and almost no neonates are admitted to hospitals when they are ill, the highest rate of neonatal mortality occurs. Neonatal mortality can be impacted by delayed healthcare seeking. Understanding care-seeking behavior reduces possible delays and significantly enhances the health of neonates [2].

### The association between mothers’ socio-demographic characteristics and neonatal danger signs

In this study the only socio-demographic variable that showed significant association were educational level and occupation of the mothers. Mothers with JHS education were four times likely to have knowledge about neonatal danger signs as compare to mothers who had primary or no formal education. This was found to be similar to a study where women whose least educational level was JHS were more likely to know about danger signs of their babies than mothers who had no formal education [4]. Similarly studies conducted in Uganda and India showed that mothers’ education status had significant association with neonatal danger signs [12]. The findings support studies conducted in Ethiopia on neonatal danger signs also corroborated with findings from other studies that found mother’s level of education to be positively associated with good knowledge on neonatal danger signs [23].

In contrast to this study other studies found an association between other socio-demographic characteristics of mother and their knowledge on neonatal danger signs. A study found that maternal age was associated with early detection of neonatal danger signs [8]. A study also found significant association between marital status of women and their knowledge on neonatal danger signs [11].

Again mothers’ occupation had significant association with knowledge on neonatal danger signs. This supports a study stating that salary mothers are about seven times likely to determine neonatal danger signs as compared to unemployed and other private workers [19]. This is because most of the salary mothers are educated and might be able to recognize neonatal danger signs. Also another study reported similar findings where employed mothers were ten times likely to know about their neonatal danger signs compared to unemployed mothers [13].

The observed differences in the findings of this study and some studies regarding association between socio-demographic characteristics of mothers and knowledge on neonatal danger signs may be due to differences in socio-demographic characteristics of the respondents. There could be other powerful factors that influence mothers’ knowledge on neonatal danger signs that were not explored. It was expected that mother’s place of birth would have a strong effect on their knowledge on neonatal danger signs. However, this study found no significant association between where mothers deliver and knowledge on neonatal danger signs. This contradicts other findings reported in literature. According to study, mothers who gave birth at the hospital had good knowledge about neonatal danger signs and were about six times more likely to know these signs as compared to mothers who delivered at home [20].

Similarly study found that place of birth has significant association with mothers’ knowledge on neonatal danger signs. Mothers with good knowledge from the midwives had good recognition of danger signs [28]. A study in northern Ghana also found place of birth to be one of the main predictors of knowledge on neonatal danger signs [17]. The observed differences in findings might be due to the differences in power relations regarding decision on how the neonates should be cared for. In the Northern region of Ghana, mother in-laws and father in-laws exert more influence on decision regarding the care of newborns although the mother may have good knowledge on neonatal danger signs. Seeking timely medical attention for the sick newborn depends heavily on the mother’s ability to recognize danger signs in the newborn. In this study it was found that, all the mothers were able to mention some of the danger signs associated with the neonates.

### Implications of the study

Recognition of neonatal dangers among mothers sets as a foundation for the appropriate interventions for the neonates. The need to pursue studies in the area of neonatal danger signs is important as poor knowledge by parents, especially the mothers regarding neonatal danger signs could pose a threat to neonatal health [8]. The Sustainable Development Goal 3 seeks to ensure wellbeing for all of all ages and encourages health for all. This study would go a long way to improve neonatal health and help achieve the goal by 2030.

It is imperative to provide comprehensive training in the field of neonatal care for mothers. For such trainings to be effective, it is important to determine the current neonatal care practices and identify deficiencies among mothers through research like this in the study area. This will enable health workers to clearly know which areas to emphasize in educating and empowering mothers to enable them improve their health care and ultimately reduce neonatal morbidity and mortality. In terms of theory this study has established the present knowledge base as well as health seeking behaviour of mothers with neonatal danger signs, making major contributions to strategies of health education and practices of these mothers within the community. Findings from the study could assist policy makers to develop appropriate policies and interventions pertaining to neonatal health and survival. Also, stakeholders will be empowered to implement the policies to bring to the fore neonatal survival issues. The goal of the Ghana National Newborn Health Strategy and Action Plan, is to reduce neonatal mortality to 21 per 1000 live births [6]. It is hoped that the findings would inform the Regional Health Directorate and facilities to re-strategize to increase mothers’ knowledge on neonatal danger signs. Also, the findings of the study would be useful for nurse managers and health administrators to formulate policies to enhance quality health care delivery in relation to maternal and child health services.

### Limitations of the study

Reaching all of the respondents was a big challenge because some were dispersed widely across the municipality. Nevertheless, participants had three weeks to respond and return in their questionnaires. Again, due to the fact that some mothers had to spend so much time caring for these babies at the Neonatal Intensive Unit (NICU) because of their ill health, the study was unable to evaluate the mothers at the intensive care units or the neonatal care units.

## Conclusions

In general, knowledge on neonatal danger signs was good among the study participants. The study found good health seeking behaviour of mothers attending health facilities on their own volition. With the association between socio-demographic characteristics of the mothers and the knowledge on neonatal danger signs, educational level and occupation of mothers were statistically significant. However, there may be other factors that influence mothers’ knowledge on neonatal danger signs that could be explored. The study therefore recommends the following:

Community Health Nurses and Midwives should follow up by home visits and encourage mothers to practice the knowledge and skills they have acquired during counseling to detect danger signs in their children. There is also the need for health managers in the municipality to develop strategies to encourage mothers to deliver at the health facility where appropriate neonatal care and counselling are provided. Mothers should be empowered to make decisions concerning their children’s health care. Further studies should be conducted to explore other household and community factors that could influence mother’s knowledge on neonatal danger signs.

### Electronic supplementary material

Below is the link to the electronic supplementary material.

Supplementary Material 1

Supplementary Material 2

## Data Availability

Data analyzed during this study may be available from the corresponding author upon reasonable request.

## Abbreviations

CHPS: Community Based Health Planning and services
HIV: Human Imuno-deficiency Virus
ICU: Intensive Care Unit
SPSS: Statistical Package of Social Sciences
TBA: Traditional Birth Attendant
WHO: World Health Organisation

## References

[1] Adigun AS. New Born Care practices and knowledge of risk factors Associated with neonatal mortality among Post Natal Mothers in Ibadan, 2018; 11(2), 1050–8.

[2] Abdullah A, Hort K, Butu Y, Simpson L. Risk factors associated with neonatal deaths: a matched case – control study in Indonesia. 2020; 1–24. 10.3402/gha.v9.30445 PMC4759830 26895147

[3] Amolo L, Irimu G, Njai D. Knowledge of postnatal mothers on essential newborn care practices at the Kenyatta National Hospital: a cross sectional study, 2017; 8688, 1–7. 10.11604/pamj.2017.28.97.13785 PMC5724942 29255567

[4] Nusrat K, Khan MR, Waseem Z, Siddiqui OM, Mahmood S. Neonatal danger signs and healthcare seeking behaviours: a cross-sectional study in Karachi amongst pregnant females. 2020; 70(1), 74–9. 10.5455/JPMA.3145 31954027

[5] Kaur H, Joshi P, Kaur M, Dhital R, Silwal RC, Simkhada P, Asfaw M. Assessing knowledge and behavioural changes on maternal and newborn health among mothers following post- Earthquake health promotion in Nepal. 2020; 34(1), 1–11. 10.6084/m9.figshare.8869337.Funding 10.1371/journal.pone.0220191 PMC6657877 31344147

[6] Masaoud A, Hussein AA, Ahmad ER. Factors associated with neonatal danger signs among high risk mothers during perinatal period. 2019; 9(4), 17–28. 10.5430/jnep.v9n4p17

[7] Alemayehu L, Irimu G, Njai D. Knowledge of postnatal mothers on essential newborn care practices at the Ethiopian National Hospital: a cross sectional study, 2019; 8688, 1–7. 10.11604/pamj.2017.28.97.13785 PMC5724942 29255567

[8] Atuyambe E, Zupan J. Neonatal and perinatal mortality: Country, regional and global estimates. 2018; 34–78.

[9] Awasthi T, Verma G, Agarwal A (2016). Newborn Care Practice and Associated Factors among mothers who gave birth within one year in Mandura District. Northwest India Clinics in Mother and Child Health.

[10] Bannerman N, Bah R, Mazumdars, Martines J, Black R, Bhan M. Effect of community –based promotion of exclusive breastfeeding on diarrhoeal Illness and growth: a Custer randomized controlled trail, Lancet 2019; 89–152. 10.1016/S0140-6736(03)13134-0 12727395

[11] Begum H, Faizul M, Khan H, Review SL. (2019). Socio-Economic Factors and Knowledge Influencing Newborn Care Practices: Experience at Dhaka Shishu Hospital. 2019; 233–288.

[12] Bello AS, Adedokun AS, Ojengbede S. New Born Care practices and knowledge of risk factors Associated with neonatal mortality among Post Natal Mothers in Ibadan, 2019; *11*(2), 1050–8.

[13] Bhandari N, Paudyal MJ. *Effect of community –based promotion of exclusive breastfeeding on diarrhoeal illness and growth*: a Custer randomized controlled trail, Lancet 2016; 145–189. 10.1016/S0140-6736(03)13134-0 12727395

[14] Callaghan-koru JA, Seifu A, Tholandi M, Graft-johnson JD, Daniel E, Rawlins B, Baqui AH. (2018). *Newborn care practices at home and in health facilities in 4 regions of Ethiopia* 10.1186/1471-2431-13-198 PMC4219496 24289501

[15] Chou SC, Palmer RH, Ezhuthachan S, Newman C, Pradell-Boyd B, Maisels MJ. (2017) *Management of hyperbilirubinemia in newborns*: measuring performance by using a benchmarking model. Pediatrics. 2017;112:1264-73. 10.1542/peds.112.6.1264 14654595

[16] Chaudhary J, Dhungana GP, Ghimire H. (2017). *Factors Affecting Newborn Care Practices Among*: Tharu Mothers in Selected Vilalge Development Committees of Chitwan Distract (August), 1–5.

[17] Clarkson JE, Cowan JO, Herbison GP (2015). Jaundice in full term healthy neonates–a population study. Aust Paediatrics Journal.

[18] Dennery PA, Seidman DS, Stevenson DK (2016). Neonatal Hyperbilirubinemia England Journal of Medicine.

[19] World Health Organisation. Essential newborn care. Report of a technical Working group Geneva. (2020); 25–29.

[20] World health organization (2019). Neonatal and perinatal mortality. World Health Organisation.

[21] Ekwochi U. Knowledge of danger signs in newborns and health seeking practices of mothers and care givers in Enugu state, South-East Nigeria. 2020; 1–16. 10.1186/s13052-015-0127-5 PMC4372313 25888409

[22] Essel E, Amenga-etego LN, Quaye SL. Int J Health Sci Res. 2018; 98–127.

[23] Ekwochi M, Ndu F, Osuorah A, Amadi F, Okeke B, Obuoha A, Anyim N (2015). Assessment of inpatient paediatric care in first referral level hospitals in 13 districts in Kenya. The Lancet.

[24] Ekwochi M, Mariama N, Ibrahim. Home birth in women who have given birth at least once in a health facility: the contributing factors in developing countries. Acta Obstet Gynaecol. 2019; 23–69 Scandinavia E publication.

[25] Engender Health. (2016). COPE handbook: a process for improving quality in health services. United States of America, 2016; 69–98.

[26] Gartner LM, Lee KS (2019). Jaundice in the breastfed infant. Clin Perinatol.

[27] Ghana Health Service. Annual Report. 2019; 23–56.

[28] Gul LC, Chirwa E, Malata A, Odland JO, Bjune G (2018). Do Malawian women critically asses the quality of care? A qualitative study on women’s perception of perinatal care at a district hospital in Malawi. Reproductive Health.

[29] Huang MJ, Kua KE, Teng HC, Tang KS, Weng HW, Huang CS (2018). Risk factors for severe hyperbilirubinemia in neonates. Pediatr Res.

[30] Jiji Z, Ha A, Soremekun S, Weobong B, Gyan T, Kirkwood BR. Increasing access to care for sick newborns: evidence from the Ghana Newhints cluster-randomised controlled trial, 2017; *6–35*. 10.1136/bmjopen-2015-008107 PMC4916576 27297006

[31] Kaphle HP, Yadav DK, Neupane N, Sharma B. (2016). Newborn Care Practices in Rural Communities of Nawalparasi District, Nepal Newborn Care Practices in Rural Communities 2018; 56–101.

[32] Kayom V, Kakuru JC, Kiguli A (2020). Neonatal Hypothermia in low resources settings: a review. J Perinatol.

[33] Kayom V, Shearer JC, Kumar A, Darmstadt GL. (2015). Neonatal hypothermia in low resources settings: A review. Journal of Perinatology, 2018; 29(6), 401–412. 10.1038/jp.2008.233 19158799

[34] Kesterton HP, Cleland N. (2019). Newborn Care Practices in Rural Communities of Nawalparasi District, Nepal Newborn Care Practices in Rural Communities of Nepal, 2019; 67–119.

[35] KHanal K, Adhikari E, Karkee A. Ghana’s Ensure mothers and Babies Regular Access to Cure (EMBRACE) program: study protocol for a cluster randomized controlled trial. Trails. 2019; 16:22. 10.1186/s/3063-034-0539-3 PMID:25887849. 10.1186/s13063-014-0539-3 PMC4324027 25887849

[36] Kibaru S (2018). Poor newborn care practices - a population based survey in eastern Uganda. BMC Pregnancy Childbirth.

[37] Kumbani LC, Chirwa E, Malata A, Odland JO, Bjune G (2019). Do Malawian women critically asses the quality of care? A qualitative study on women’s perception of perinatal care at a district hospital in Malawi. Reproductive Health.

[38] Kuyanab P, Yem HC, Yidana A (2017). Neonatal Hypothermia in low resources settings: a review. J Perinatol.

[39] Lee ET, Vientiane AF, Durham CB, Booth RT, Sychareum O. Three decades after Alma –Ata: are women satisfied with Antenatal Care Services at Primary Health Centres in Mushin, Lagos? Online Journal of Medicine and Medical Science Research 2019; (3),29.

[40] Mbwele B, Reddy E, Reyburn H. (2018). *A rapid assessment of the quality of neonatal healthcare in Kilimanjaro region*, Northeast Tanzania BMC Pediatr.2013,12(1):182. 10.1186/1471-2431-12-182 PMC3542091 23171226

[41] Misgna HG, Gebru HB, Birhanu MM. Knowledge, practice and associated factors of essential newborn care at home among mothers in Gulomekada District, Eastern. BMC Pregnancy Childbirth, 2017. 1–8. 10.1186/s12884-016-0931-y PMC4915039 27329372

[42] Nepal HG, Joshi HB, Sharma MM, Teijingen P. Knowledge, practice and associated factors of essential newborn care at home among mothers in Gulomekada District, Eastern. BMC Pregnancy Childbirth, 2018; 1–8. 10.1186/s12884-016-0931-y PMC4915039 27329372

[43] Nigatu SG, Worku AG, Dadi AF. Level of mother’s knowledge about neonatal danger signs and associated factors in North West of Ethiopia: a community based study. BMC Res Notes, 2015; 4–9. 10.1186/s13104-015-1278-6 PMC4506763 26188481

[44] Padiyath DA, Bhat PR, Ekambaram BP. Trends and determinants of neonatal mortality in Nepal, -2019; 45–79.

[45] UNICEF. The State of World Children. 2017; 10.4172/2090-4.1000172

