# Supplementary material for: Health seeking behaviour and knowledge on neonatal danger signs among neonatal caregivers in Upper Denkyira East Municipality, Ghana
Source: BMC Pediatr. 2024 Jan 8;24:27. doi: 10.1186/s12887-023-04430-2 (PMC10773034; doi:10.1186/s12887-023-04430-2)
Supplement: Supplementary file 1 — Supplementary Material 1 [file 12887_2023_4430_MOESM1_ESM.pdf]

Age	Marital	Religion	Tribe	Education	occupation	Delivery	Breastfeeding	Mixed
3.00	2	2	1	5	4	1	1	99
2.00	2	1	1	5	4	1	1	99
3.00	2	2	4	5	4	2	1	99
2.00	2	2	1	3	4	1	2	3
3.00	2	2	1	3	3	1	1	99
2.00	1	1	1	2	1	1	2	1
3.00	2	2	2	3	3	1	2	3
2.00	2	2	2	3	2	1	1	99
3.00	1	1	2	2	2	1	1	99
2.00	3	2	2	4	5	1	1	99
1.00	1	1	1	1	1	1	1	99
2.00	1	1	1	4	1	1	1	99
2.00	3	2	1	5	4	1	1	99
2.00	1	2	1	1	1	1	1	99
2.00	2	1	4	5	4	1	1	99
1.00	1	1	1	1	3	1	2	2
3.00	2	2	1	4	3	1	1	99
2.00	1	1	1	1	1	1	1	99
3.00	3	2	1	5	4	1	1	99
2.00	3	2	3	5	4	1	1	99
2.00	2	1	4	1	3	1	1	99
1.00	3	2	1	3	2	1	2	2
2.00	3	1	1	2	4	1	1	99
2.00	2	2	3	5	4	1	2	2
2.00	1	2	1	3	3	1	2	1
2.00	2	2	3	5	4	1	1	99
3.00	3	2	1	2	2	1	2	1
3.00	1	1	1	3	4	1	1	99
3.00	2	2	3	4	4	1	1	99
2.00	2	1	1	5	4	1	1	99
1.00	1	2	1	4	5	1	2	2
3.00	2	1	1	5	4	1	1	99
3.00	2	2	1	5	3	1	1	99
2.00	2	2	3	5	4	1	1	99
2.00	2	1	1	3	4	1	1	99
2.00	2	1	2	3	3	2	2	1
2.00	1	1	1	1	1	1	2	3
3.00	2	1	3	5	4	1	1	99
1.00	1	1	4	4	5	1	2	1
1.00	1	1	1	3	5	1	1	99
2.00	3	2	1	4	2	1	1	99
2.00	2	2	1	4	2	1	1	99
2.00	1	2	2	5	4	1	1	99
2.00	2	2	1	5	3	1	2	1
3.00	2	1	1	5	4	1	1	99
2.00	3	1	1	3	2	2	2	1

2.00	2	1	4	2	2	1	1	99
1.00	1	2	1	4	2	1	2	2
3.00	2	2	1	5	4	1	1	99
1.00	1	2	1	3	2	2	2	1
3.00	2	1	4	4	2	1	2	2
1.00	3	2	1	3	2	1	2	1
3.00	3	1	1	2	3	1	2	1
3.00	2	2	3	5	4	1	1	99
2.00	2	2	2	5	4	1	2	2
2.00	2	2	1	3	2	1	2	2
1.00	3	1	3	5	4	2	1	99
3.00	2	2	3	3	2	1	1	99
3.00	3	2	2	5	4	1	1	99
2.00	1	2	1	4	2	1	1	99
3.00	3	2	1	4	3	1	2	1
1.00	1	1	1	3	5	1	2	1
1.00	2	2	1	4	2	1	1	99
3.00	2	2	3	4	1	1	2	2
1.00	1	2	4	3	5	2	2	1
2.00	3	1	4	5	4	1	1	99
2.00	1	2	1	2	1	1	1	99
2.00	2	2	2	5	4	1	1	99
1.00	1	2	1	4	5	1	2	2
1.00	1	2	1	4	2	1	2	2
1.00	3	1	1	4	2	1	2	2
2.00	3	1	1	5	4	1	2	2
3.00	2	2	1	5	4	1	1	99
2.00	2	1	1	1	1	1	2	1
2.00	2	2	1	4	2	1	1	99
2.00	2	1	2	4	2	1	2	2
3.00	1	2	1	3	4	1	1	99
2.00	2	1	1	4	2	1	2	2
2.00	2	1	1	3	1	1	1	99
2.00	1	2	1	3	1	1	1	99
2.00	2	1	1	5	4	1	2	2
3.00	2	2	1	5	4	1	1	99
2.00	3	1	4	3	2	1	1	99
2.00	3	2	1	4	2	1	1	99
2.00	2	2	1	5	4	1	1	99
2.00	3	2	2	5	4	1	1	99
1.00	2	1	3	4	5	2	2	3
1.00	1	1	4	3	5	1	2	1
2.00	2	1	1	3	1	1	1	99
2.00	2	1	2	5	4	1	2	2
2.00	2	1	1	5	4	1	2	2
2.00	2	2	2	5	4	1	1	99
3.00	3	2	2	5	4	1	1	99

2.00	1	1	1	4	1	1	2	1
2.00	3	2	1	3	2	1	1	99
2.00	2	1	4	5	4	1	1	99
3.00	3	2	2	5	4	1	1	99
3.00	2	2	1	4	3	1	2	1
2.00	3	2	1	4	1	1	1	99
3.00	2	2	1	5	2	1	1	99
2.00	2	2	1	4	5	2	1	99
1.00	1	1	1	2	5	2	2	3
1.00	1	1	4	5	5	2	2	1
3.00	2	2	1	3	1	1	1	99
2.00	2	2	1	4	5	1	1	99
2.00	1	2	1	5	4	1	1	99
3.00	2	2	1	5	4	1	1	99
2.00	1	2	1	5	5	1	1	99
3.00	1	2	1	4	3	1	1	99
2.00	2	2	1	1	5	1	2	1
3.00	2	2	1	1	4	1	1	99
2.00	2	2	4	4	1	1	1	99
2.00	1	2	1	5	4	1	1	99
1.00	1	2	2	3	5	1	2	1
3.00	2	2	1	2	4	1	2	1
2.00	2	2	1	3	1	1	1	99
2.00	2	2	1	4	5	1	1	99
2.00	2	2	1	3	5	1	1	99
2.00	1	2	1	5	4	1	1	99
2.00	2	2	1	5	4	1	1	99
2.00	2	2	1	4	3	1	1	99
2.00	2	2	1	4	5	2	2	1
3.00	2	2	1	5	3	1	1	99
3.00	1	2	1	2	1	1	1	99
2.00	4	2	1	1	1	1	1	99
3.00	2	2	1	5	4	1	1	99
2.00	2	1	1	5	4	1	1	99
3.00	2	2	4	5	4	2	1	99
2.00	2	2	1	3	4	1	2	3
3.00	2	2	1	3	3	1	1	99
2.00	1	1	1	2	1	1	2	1
3.00	2	2	2	3	3	1	2	3
2.00	2	2	2	3	2	1	1	99
3.00	1	1	2	2	2	1	1	99
2.00	3	2	2	4	5	1	1	99
1.00	1	1	1	1	1	1	1	99
2.00	1	1	1	4	1	1	1	99
2.00	3	2	1	5	4	1	1	99
2.00	1	2	1	1	1	1	1	99
2.00	2	1	4	5	4	1	1	99

1.00	1	1	1	1	3	1	2	2
3.00	2	2	1	4	3	1	1	99
2.00	1	1	1	1	1	1	1	99
3.00	3	2	1	5	4	1	1	99
2.00	3	2	3	5	4	1	1	99
2.00	2	1	4	1	3	1	1	99
1.00	3	2	1	3	2	1	2	2
2.00	3	1	1	2	4	1	1	99
2.00	2	2	3	5	4	1	2	2
2.00	1	2	1	3	3	1	2	1
2.00	2	2	3	5	4	1	1	99
3.00	3	2	1	2	2	1	2	1
3.00	1	1	1	3	4	1	1	99
3.00	2	2	3	4	4	1	1	99
2.00	2	1	1	5	4	1	1	99
1.00	1	2	1	4	5	1	2	2
3.00	2	1	1	5	4	1	1	99
3.00	2	2	1	5	3	1	1	99
2.00	2	2	3	5	4	1	1	99
2.00	2	1	1	3	4	1	1	99
2.00	2	1	2	3	3	2	2	1
2.00	1	1	1	1	1	1	2	3
3.00	2	1	3	5	4	1	1	99
1.00	1	1	4	4	5	1	2	1
1.00	1	1	1	3	5	1	1	99
2.00	3	2	1	4	2	1	1	99
2.00	2	2	1	4	2	1	1	99
2.00	1	2	2	5	4	1	1	99
2.00	2	2	1	5	3	1	2	1
3.00	2	1	1	5	4	1	1	99
2.00	3	1	1	3	2	2	2	1
2.00	2	1	4	2	2	1	1	99
1.00	1	2	1	4	2	1	2	2
3.00	2	2	1	5	4	1	1	99
1.00	1	2	1	3	2	2	2	1
3.00	2	1	4	4	2	1	2	2
1.00	3	2	1	3	2	1	2	1
3.00	3	1	1	2	3	1	2	1
3.00	2	2	3	5	4	1	1	99
2.00	2	2	2	5	4	1	2	2
2.00	2	2	1	3	2	1	2	2
1.00	3	1	3	5	4	2	1	99
3.00	2	2	3	3	2	1	1	99
3.00	3	2	2	5	4	1	1	99
2.00	1	2	1	4	2	1	1	99
3.00	3	2	1	4	3	1	2	1
1.00	1	1	1	3	5	1	2	1

1.00	2	2	1	4	2	1	1	99
3.00	2	2	3	4	1	1	2	2
1.00	1	2	4	3	5	2	2	1
2.00	3	1	4	5	4	1	1	99
2.00	1	2	1	2	1	1	1	99
2.00	2	2	2	5	4	1	1	99
1.00	1	2	1	4	5	1	2	2
1.00	1	2	1	4	2	1	2	2
1.00	3	1	1	4	2	1	2	2
2.00	3	1	1	5	4	1	2	2
3.00	2	2	1	5	4	1	1	99
2.00	2	1	1	1	1	1	2	1
2.00	2	2	1	4	2	1	1	99
2.00	2	1	2	4	2	1	2	2
3.00	1	2	1	3	4	1	1	99
2.00	2	1	1	4	2	1	2	2
2.00	2	1	1	3	1	1	1	99
2.00	1	2	1	3	1	1	1	99
2.00	2	1	1	5	4	1	2	2
3.00	2	2	1	5	4	1	1	99
2.00	3	1	4	3	2	1	1	99
2.00	3	2	1	4	2	1	1	99
2.00	2	2	1	5	4	1	1	99
2.00	3	2	2	5	4	1	1	99
1.00	2	1	3	4	5	2	2	3
1.00	1	1	4	3	5	1	2	1
2.00	2	1	1	3	1	1	1	99
2.00	2	1	2	5	4	1	2	2
2.00	2	1	1	5	4	1	2	2
2.00	2	2	2	5	4	1	1	99
3.00	3	2	2	5	4	1	1	99
2.00	1	1	1	4	1	1	2	1
2.00	3	2	1	3	2	1	1	99
2.00	2	1	4	5	4	1	1	99
3.00	3	2	2	5	4	1	1	99
3.00	2	2	1	4	3	1	2	1
2.00	3	2	1	4	1	1	1	99
3.00	2	2	1	5	2	1	1	99
2.00	2	2	1	4	5	2	1	99
1.00	1	1	1	2	5	2	2	3
1.00	1	1	4	5	5	2	2	1
3.00	2	2	1	3	1	1	1	99
2.00	2	2	1	4	5	1	1	99
2.00	1	2	1	5	4	1	1	99
3.00	2	2	1	5	4	1	1	99
2.00	1	2	1	5	5	1	1	99
3.00	1	2	1	4	3	1	1	99

2.00	2	2	1	1	5	1	2	1
3.00	2	2	1	1	4	1	1	99
2.00	2	2	4	4	1	1	1	99
2.00	1	2	1	5	4	1	1	99
1.00	1	2	2	3	5	1	2	1
3.00	2	2	1	2	4	1	2	1
2.00	2	2	1	3	1	1	1	99
2.00	2	2	1	4	5	1	1	99
2.00	2	2	1	3	5	1	1	99
2.00	1	2	1	5	4	1	1	99
2.00	2	2	1	5	4	1	1	99
2.00	2	2	1	4	3	1	1	99
2.00	2	2	1	4	5	2	2	1
3.00	2	2	1	5	3	1	1	99
3.00	1	2	1	2	1	1	1	99
2.00	4	2	1	1	1	1	1	99
3.00	2	2	1	5	4	1	1	99
2.00	2	1	1	5	4	1	1	99
3.00	2	2	4	5	4	2	1	99
2.00	2	2	1	3	4	1	2	3
3.00	2	2	1	3	3	1	1	99
2.00	1	1	1	2	1	1	2	1
3.00	2	2	2	3	3	1	2	3
2.00	2	2	2	3	2	1	1	99
3.00	1	1	2	2	2	1	1	99
2.00	3	2	2	4	5	1	1	99
1.00	1	1	1	1	1	1	1	99
2.00	1	1	1	4	1	1	1	99
2.00	3	2	1	5	4	1	1	99
2.00	1	2	1	1	1	1	1	99
2.00	2	1	4	5	4	1	1	99
1.00	1	1	1	1	3	1	2	2
3.00	2	2	1	4	3	1	1	99
2.00	1	1	1	1	1	1	1	99
3.00	3	2	1	5	4	1	1	99
2.00	3	2	3	5	4	1	1	99
2.00	2	1	4	1	3	1	1	99
1.00	3	2	1	3	2	1	2	2
2.00	3	1	1	2	4	1	1	99
2.00	2	2	3	5	4	1	2	2
2.00	1	2	1	3	3	1	2	1
2.00	2	2	3	5	4	1	1	99
3.00	3	2	1	2	2	1	2	1
3.00	1	1	1	3	4	1	1	99
3.00	2	2	3	4	4	1	1	99
2.00	2	1	1	5	4	1	1	99
1.00	1	2	1	4	5	1	2	2

3.00	2	1	1	5	4	1	1	99
3.00	2	2	1	5	3	1	1	99
2.00	2	2	3	5	4	1	1	99
2.00	2	1	1	3	4	1	1	99
2.00	2	1	2	3	3	2	2	1
2.00	1	1	1	1	1	1	2	3
3.00	2	1	3	5	4	1	1	99
1.00	1	1	4	4	5	1	2	1
1.00	1	1	1	3	5	1	1	99
2.00	3	2	1	4	2	1	1	99
2.00	2	2	1	4	2	1	1	99
2.00	1	2	2	5	4	1	1	99
2.00	2	2	1	5	3	1	2	1
3.00	2	1	1	5	4	1	1	99
2.00	3	1	1	3	2	2	2	1
2.00	2	1	4	2	2	1	1	99
1.00	1	2	1	4	2	1	2	2
3.00	2	2	1	5	4	1	1	99
1.00	1	2	1	3	2	2	2	1
3.00	2	1	4	4	2	1	2	2
1.00	3	2	1	3	2	1	2	1
3.00	3	1	1	2	3	1	2	1
3.00	2	2	3	5	4	1	1	99
2.00	2	2	2	5	4	1	2	2
2.00	2	2	1	3	2	1	2	2
1.00	3	1	3	5	4	2	1	99
3.00	2	2	3	3	2	1	1	99
3.00	3	2	2	5	4	1	1	99
2.00	1	2	1	4	2	1	1	99
3.00	3	2	1	4	3	1	2	1
1.00	1	1	1	3	5	1	2	1
1.00	2	2	1	4	2	1	1	99
3.00	2	2	3	4	1	1	2	2
1.00	1	2	4	3	5	2	2	1
2.00	3	1	4	5	4	1	1	99
2.00	1	2	1	2	1	1	1	99
2.00	2	2	2	5	4	1	1	99
1.00	1	2	1	4	5	1	2	2
1.00	1	2	1	4	2	1	2	2
1.00	3	1	1	4	2	1	2	2
2.00	3	1	1	5	4	1	2	2
3.00	2	2	1	5	4	1	1	99
2.00	2	1	1	1	1	1	2	1
2.00	2	2	1	4	2	1	1	99
2.00	2	1	2	4	2	1	2	2
3.00	1	2	1	3	4	1	1	99
2.00	2	1	1	4	2	1	2	2

2.00	2	1	1	3	1	1	1	99
2.00	1	2	1	3	1	1	1	99
2.00	2	1	1	5	4	1	2	2
3.00	2	2	1	5	4	1	1	99
2.00	3	1	4	3	2	1	1	99
2.00	3	2	1	4	2	1	1	99
2.00	2	2	1	5	4	1	1	99
2.00	3	2	2	5	4	1	1	99
1.00	2	1	3	4	5	2	2	3
1.00	1	1	4	3	5	1	2	1
2.00	2	1	1	3	1	1	1	99
2.00	2	1	2	5	4	1	2	2
2.00	2	1	1	5	4	1	2	2
2.00	2	2	2	5	4	1	1	99
3.00	3	2	2	5	4	1	1	99
2.00	1	1	1	4	1	1	2	1
2.00	3	2	1	3	2	1	1	99
2.00	2	1	4	5	4	1	1	99
3.00	3	2	2	5	4	1	1	99
3.00	2	2	1	4	3	1	2	1
2.00	3	2	1	4	1	1	1	99
3.00	2	2	1	5	2	1	1	99
2.00	2	2	1	4	5	2	1	99
1.00	1	1	1	2	5	2	2	3
1.00	1	1	4	5	5	2	2	1
3.00	2	2	1	3	1	1	1	99
2.00	2	2	1	4	5	1	1	99
2.00	1	2	1	5	4	1	1	99
3.00	2	2	1	5	4	1	1	99
2.00	1	2	1	5	5	1	1	99
3.00	1	2	1	4	3	1	1	99
2.00	2	2	1	1	5	1	2	1
3.00	2	2	1	1	4	1	1	99
2.00	2	2	4	4	1	1	1	99
2.00	1	2	1	5	4	1	1	99
1.00	1	2	2	3	5	1	2	1
3.00	2	2	1	2	4	1	2	1
2.00	2	2	1	3	1	1	1	99
2.00	2	2	1	4	5	1	1	99
2.00	2	2	1	3	5	1	1	99
2.00	1	2	1	5	4	1	1	99
2.00	2	2	1	5	4	1	1	99
2.00	2	2	1	4	3	1	1	99
2.00	2	2	1	4	5	2	2	1
3.00	2	2	1	5	3	1	1	99
3.00	1	2	1	2	1	1	1	99
2.00	4	2	1	1	1	1	1	99

3.00	2	2	1	5	4	1	1	99
2.00	2	1	1	5	4	1	1	99
3.00	2	2	4	5	4	2	1	99
2.00	2	2	1	3	4	1	2	3
3.00	2	2	1	3	3	1	1	99
2.00	1	1	1	2	1	1	2	1

Baby	No	Yellowish	Feed	Well	Cord	Bleeding	Medication	Wash
1	99	1	1	1	1	3	1	2
1	99	1	1	2	1	3	2	1
1	99	1	1	1	1	3	2	1
1	99	2	1	1	1	3	2	2
1	99	1	1	2	1	3	1	2
1	99	1	1	1	2	3	1	3
1	99	1	1	2	1	3	1	2
1	99	1	1	2	2	3	1	2
1	99	1	1	1	1	2	1	2
1	99	1	1	4	1	3	1	2
1	99	2	1	1	1	3	1	2
1	99	1	1	2	2	3	1	2
1	99	1	1	4	1	3	1	1
1	99	1	1	2	1	3	1	2
1	99	1	1	1	1	3	2	2
1	99	1	1	4	2	3	1	1
1	99	1	1	2	1	3	1	2
1	99	2	1	1	1	3	1	2
1	99	2	1	2	1	3	1	1
1	99	1	1	1	3	2	2	3
1	99	1	1	2	1	3	1	2
1	99	1	1	2	1	4	1	2
1	99	2	1	1	1	3	1	2
1	99	1	1	2	1	3	1	2
1	99	1	1	3	1	4	1	2
1	99	1	1	1	1	3	1	3
1	99	2	1	1	2	3	1	2
1	99	1	1	1	1	3	1	2
1	99	1	1	1	1	3	1	2
1	99	1	1	2	1	3	2	2
1	99	2	1	2	2	4	1	2
1	99	1	1	1	2	3	1	2
1	99	1	1	2	2	4	1	1
1	99	1	1	2	1	3	1	2
1	99	1	1	1	1	3	1	2
1	99	1	1	3	1	3	1	2
1	99	1	1	2	1	3	1	2
1	99	1	1	2	1	3	1	2
2	1	1	1	3	2	4	1	2
1	99	1	1	1	2	3	1	2
1	99	1	1	2	1	4	1	1
1	99	1	1	1	1	3	1	2
1	99	1	1	1	1	3	1	1
1	99	1	1	1	1	3	1	1
1	99	1	1	1	1	3	1	2
1	99	1	1	2	1	3	3	2

1	99	1	1	2	1	3	1	1
1	99	1	1	2	2	3	1	2
1	99	1	1	1	2	3	1	4
1	99	2	1	2	2	3	1	2
1	99	1	1	1	1	3	1	2
1	99	1	1	2	1	4	1	2
1	99	1	1	1	2	3	1	2
1	99	2	1	1	2	3	1	2
1	99	1	1	1	1	4	1	4
1	99	1	1	2	1	3	1	2
1	99	1	1	1	1	4	1	2
1	99	1	1	2	1	3	1	2
1	99	1	1	2	3	3	2	2
1	99	2	1	1	2	4	1	2
1	99	1	1	1	1	4	1	2
1	99	1	1	2	3	1	1	3
1	99	1	1	2	2	3	1	2
1	99	1	1	1	1	3	1	1
1	99	1	1	1	1	3	1	2
1	99	1	1	2	1	3	1	1
1	99	1	1	2	1	3	1	1
1	99	1	1	1	2	3	1	3
1	99	1	1	1	1	4	1	1
1	99	1	1	2	1	3	1	1
1	99	1	1	1	1	3	1	3
1	99	1	1	2	1	3	1	2
1	99	1	1	2	1	3	2	1
1	99	1	1	2	1	3	1	2
1	99	1	1	2	3	1	1	2
1	99	1	1	2	1	4	1	2
1	99	1	1	1	1	3	1	2
1	99	1	1	2	1	4	1	2
1	99	1	1	1	2	3	1	1
1	99	1	1	1	1	4	1	2
1	99	1	1	1	1	4	1	2
1	99	1	1	2	1	3	1	2
1	99	1	1	2	1	3	1	2
1	99	1	1	1	2	3	1	2
1	99	1	1	2	2	3	2	1
1	99	1	1	1	1	4	2	2
1	99	1	1	2	1	4	1	2
1	99	1	1	1	2	3	3	2
1	99	1	1	1	2	3	1	2
1	99	1	1	1	2	3	2	2
1	99	1	1	1	2	3	1	2
1	99	1	1	1	2	1	4	1
1	99	1	1	1	1	4	1	2

1	99	1	1	2	1	4	1	1
1	99	1	1	2	1	3	1	2
1	99	1	1	1	1	4	1	3
1	99	1	1	1	1	4	1	1
1	99	1	1	3	1	3	1	1
1	99	1	1	1	3	1	1	1
1	99	1	1	1	4	4	2	1
1	99	1	1	1	2	4	1	1
1	99	1	1	4	1	4	1	2
1	99	1	1	1	1	4	1	2
1	99	1	1	1	1	4	1	1
1	99	1	1	1	2	4	1	1
1	99	1	1	1	3	3	1	1
1	99	1	1	2	1	4	1	2
1	99	1	1	1	2	4	2	2
1	99	1	1	1	3	3	1	2
2	1	1	1	4	2	4	1	2
1	99	1	1	1	1	4	1	1
1	99	1	1	1	1	1	3	1
1	99	1	1	2	1	4	1	1
1	99	1	1	3	3	1	1	2
1	99	1	1	2	1	4	1	1
1	99	1	1	2	1	4	1	2
1	99	1	1	2	1	3	1	1
1	99	1	1	3	1	3	1	1
1	99	1	1	2	3	4	1	1
1	99	1	1	1	1	3	1	1
1	99	1	1	1	1	3	1	2
1	99	2	1	2	1	3	2	3
1	99	1	1	2	2	3	1	2
1	99	1	1	2	2	3	1	2
1	99	1	1	2	1	3	1	2
1	99	1	1	1	1	3	1	2
1	99	1	1	2	1	3	2	1
1	99	1	1	1	1	3	2	1
1	99	2	1	1	1	3	2	2
1	99	1	1	2	1	3	1	2
1	99	1	1	1	2	3	1	3
1	99	1	1	2	1	3	1	2
1	99	1	1	2	2	3	1	2
1	99	1	1	1	1	2	1	2
1	99	1	1	4	1	3	1	2
1	99	2	1	1	1	3	1	2
1	99	1	1	2	2	3	1	2
1	99	1	1	4	1	3	1	1
1	99	1	1	2	1	3	1	2
1	99	1	1	1	1	3	2	2

1	99	1	1	4	2	3	1	1
1	99	1	1	2	1	3	1	2
1	99	2	1	1	1	3	1	2
1	99	2	1	2	1	3	1	1
1	99	1	1	1	3	2	2	3
1	99	1	1	2	1	3	1	2
1	99	1	1	2	1	4	1	2
1	99	2	1	1	1	3	1	2
1	99	1	1	2	1	3	1	2
1	99	1	1	3	1	4	1	2
1	99	1	1	1	1	3	1	3
1	99	2	1	1	2	3	1	2
1	99	1	1	1	1	3	1	2
1	99	1	1	1	1	3	1	2
1	99	1	1	2	1	3	2	2
1	99	2	1	2	2	4	1	2
1	99	1	1	1	2	3	1	2
1	99	1	1	2	2	4	1	1
1	99	1	1	2	1	3	1	2
1	99	1	1	1	1	3	1	2
1	99	1	1	3	1	3	1	2
1	99	1	1	2	1	3	1	2
1	99	1	1	2	1	3	1	2
2	1	1	1	3	2	4	1	2
1	99	1	1	1	2	3	1	2
1	99	1	1	2	1	4	1	1
1	99	1	1	1	1	3	1	2
1	99	1	1	1	1	3	1	1
1	99	1	1	1	1	3	1	1
1	99	1	1	1	1	3	1	2
1	99	1	1	2	1	3	3	2
1	99	1	1	2	1	3	1	1
1	99	1	1	2	2	3	1	2
1	99	1	1	1	2	3	1	4
1	99	2	1	2	2	3	1	2
1	99	1	1	1	1	3	1	2
1	99	1	1	2	1	4	1	2
1	99	1	1	1	2	3	1	2
1	99	2	1	1	2	3	1	2
1	99	1	1	1	1	4	1	4
1	99	1	1	2	1	3	1	2
1	99	1	1	1	1	4	1	2
1	99	1	1	2	1	3	1	2
1	99	1	1	2	3	3	2	2
1	99	2	1	1	2	4	1	2
1	99	1	1	1	1	4	1	2
1	99	1	1	2	3	1	1	3

1	99	1	1	2	2	3	1	2
1	99	1	1	1	1	3	1	1
1	99	1	1	1	1	3	1	2
1	99	1	1	2	1	3	1	1
1	99	1	1	2	1	3	1	1
1	99	1	1	1	2	3	1	3
1	99	1	1	1	1	4	1	1
1	99	1	1	2	1	3	1	1
1	99	1	1	1	1	3	1	3
1	99	1	1	2	1	3	1	2
1	99	1	1	2	1	3	2	1
1	99	1	1	2	1	3	1	2
1	99	1	1	2	3	1	1	2
1	99	1	1	2	1	4	1	2
1	99	1	1	1	1	3	1	2
1	99	1	1	2	1	4	1	2
1	99	1	1	1	2	3	1	1
1	99	1	1	1	1	4	1	2
1	99	1	1	1	1	4	1	2
1	99	1	1	2	1	3	1	2
1	99	1	1	2	1	3	1	2
1	99	1	1	1	2	3	1	2
1	99	1	1	2	2	3	2	1
1	99	1	1	1	1	4	2	2
1	99	1	1	2	1	4	1	2
1	99	1	1	1	2	3	3	2
1	99	1	1	1	2	3	1	2
1	99	1	1	1	2	3	2	2
1	99	1	1	1	2	3	1	2
1	99	1	1	1	2	1	4	1
1	99	1	1	1	1	4	1	2
1	99	1	1	2	1	4	1	1
1	99	1	1	2	1	3	1	2
1	99	1	1	1	1	4	1	3
1	99	1	1	1	1	4	1	1
1	99	1	1	3	1	3	1	1
1	99	1	1	1	3	1	1	1
1	99	1	1	1	4	4	2	1
1	99	1	1	1	2	4	1	1
1	99	1	1	4	1	4	1	2
1	99	1	1	1	1	4	1	2
1	99	1	1	1	1	4	1	1
1	99	1	1	1	2	4	1	1
1	99	1	1	1	3	3	1	1
1	99	1	1	2	1	4	1	2
1	99	1	1	1	2	4	2	2
1	99	1	1	1	3	3	1	2

2	1	1	1	4	2	4	1	2
1	99	1	1	1	1	4	1	1
1	99	1	1	1	1	1	3	1
1	99	1	1	2	1	4	1	1
1	99	1	1	3	3	1	1	2
1	99	1	1	2	1	4	1	1
1	99	1	1	2	1	4	1	2
1	99	1	1	2	1	3	1	1
1	99	1	1	3	1	3	1	1
1	99	1	1	2	3	4	1	1
1	99	1	1	1	1	3	1	1
1	99	1	1	1	1	3	1	2
1	99	2	1	2	1	3	2	3
1	99	1	1	2	2	3	1	2
1	99	1	1	2	2	3	1	2
1	99	1	1	2	1	3	1	2
1	99	1	1	1	1	3	1	2
1	99	1	1	2	1	3	2	1
1	99	1	1	1	1	3	2	1
1	99	2	1	1	1	3	2	2
1	99	1	1	2	1	3	1	2
1	99	1	1	1	2	3	1	3
1	99	1	1	2	1	3	1	2
1	99	1	1	2	2	3	1	2
1	99	1	1	1	1	2	1	2
1	99	1	1	4	1	3	1	2
1	99	2	1	1	1	3	1	2
1	99	1	1	2	2	3	1	2
1	99	1	1	4	1	3	1	1
1	99	1	1	2	1	3	1	2
1	99	1	1	1	1	3	2	2
1	99	1	1	4	2	3	1	1
1	99	1	1	2	1	3	1	2
1	99	2	1	1	1	3	1	2
1	99	2	1	2	1	3	1	1
1	99	1	1	1	3	2	2	3
1	99	1	1	2	1	3	1	2
1	99	1	1	2	1	4	1	2
1	99	2	1	1	1	3	1	2
1	99	1	1	2	1	3	1	2
1	99	1	1	3	1	4	1	2
1	99	1	1	1	1	3	1	3
1	99	2	1	1	2	3	1	2
1	99	1	1	1	1	3	1	2
1	99	1	1	1	1	3	1	2
1	99	1	1	2	1	3	2	2
1	99	2	1	2	2	4	1	2

1	99	1	1	1	2	3	1	2
1	99	1	1	2	2	4	1	1
1	99	1	1	2	1	3	1	2
1	99	1	1	1	1	3	1	2
1	99	1	1	3	1	3	1	2
1	99	1	1	2	1	3	1	2
1	99	1	1	2	1	3	1	2
2	1	1	1	3	2	4	1	2
1	99	1	1	1	2	3	1	2
1	99	1	1	2	1	4	1	1
1	99	1	1	1	1	3	1	2
1	99	1	1	1	1	3	1	1
1	99	1	1	1	1	3	1	1
1	99	1	1	1	1	3	1	2
1	99	1	1	2	1	3	3	2
1	99	1	1	2	1	3	1	1
1	99	1	1	2	2	3	1	2
1	99	1	1	1	2	3	1	4
1	99	2	1	2	2	3	1	2
1	99	1	1	1	1	3	1	2
1	99	1	1	2	1	4	1	2
1	99	1	1	1	2	3	1	2
1	99	2	1	1	2	3	1	2
1	99	1	1	1	1	4	1	4
1	99	1	1	2	1	3	1	2
1	99	1	1	1	1	4	1	2
1	99	1	1	2	1	3	1	2
1	99	1	1	2	3	3	2	2
1	99	2	1	1	2	4	1	2
1	99	1	1	1	1	4	1	2
1	99	1	1	2	3	1	1	3
1	99	1	1	2	2	3	1	2
1	99	1	1	1	1	3	1	1
1	99	1	1	1	1	3	1	2
1	99	1	1	2	1	3	1	1
1	99	1	1	2	1	3	1	1
1	99	1	1	1	2	3	1	3
1	99	1	1	1	1	4	1	1
1	99	1	1	2	1	3	1	1
1	99	1	1	1	1	3	1	3
1	99	1	1	2	1	3	1	2
1	99	1	1	2	1	3	2	1
1	99	1	1	2	1	3	1	2
1	99	1	1	2	3	1	1	2
1	99	1	1	2	1	4	1	2
1	99	1	1	1	1	3	1	2
1	99	1	1	2	1	4	1	2

1	99	1	1	1	2	3	1	1
1	99	1	1	1	1	4	1	2
1	99	1	1	1	1	4	1	2
1	99	1	1	2	1	3	1	2
1	99	1	1	2	1	3	1	2
1	99	1	1	1	2	3	1	2
1	99	1	1	2	2	3	2	1
1	99	1	1	1	1	4	2	2
1	99	1	1	2	1	4	1	2
1	99	1	1	1	2	3	3	2
1	99	1	1	1	2	3	1	2
1	99	1	1	1	2	3	2	2
1	99	1	1	1	2	3	1	2
1	99	1	1	1	2	1	4	1
1	99	1	1	1	1	4	1	2
1	99	1	1	2	1	4	1	1
1	99	1	1	2	1	3	1	2
1	99	1	1	1	1	4	1	3
1	99	1	1	1	1	4	1	1
1	99	1	1	3	1	3	1	1
1	99	1	1	1	3	1	1	1
1	99	1	1	1	4	4	2	1
1	99	1	1	1	2	4	1	1
1	99	1	1	4	1	4	1	2
1	99	1	1	1	1	4	1	2
1	99	1	1	1	1	4	1	1
1	99	1	1	1	2	4	1	1
1	99	1	1	1	3	3	1	1
1	99	1	1	2	1	4	1	2
1	99	1	1	1	2	4	2	2
1	99	1	1	1	3	3	1	2
2	1	1	1	4	2	4	1	2
1	99	1	1	1	1	4	1	1
1	99	1	1	1	1	1	3	1
1	99	1	1	2	1	4	1	1
1	99	1	1	3	3	1	1	2
1	99	1	1	2	1	4	1	1
1	99	1	1	2	1	4	1	2
1	99	1	1	2	1	3	1	1
1	99	1	1	3	1	3	1	1
1	99	1	1	2	3	4	1	1
1	99	1	1	1	1	3	1	1
1	99	1	1	1	1	3	1	2
1	99	2	1	2	1	3	2	3
1	99	1	1	2	2	3	1	2
1	99	1	1	2	2	3	1	2
1	99	1	1	2	1	3	1	2

1	99	1	1	1	1	3	1	2
1	99	1	1	2	1	3	2	1
1	99	1	1	1	1	3	2	1
1	99	2	1	1	1	3	2	2
1	99	1	1	2	1	3	1	2
1	99	1	1	1	2	3	1	3

Conditions	Antenatal	If	Visits	Trimester	Gestational	Advise
2	1	3	2	1	1	4
1	1	1	3	2	1	4
2	1	3	4	1	1	4
2	1	2	3	3	1	4
4	1	2	3	1	1	4
7	1	3	2	1	1	4
7	1	3	3	2	1	4
1	1	2	3	1	1	4
2	2	99	99	99	1	2
1	1	2	4	2	1	1
2	2	99	99	99	1	4
7	1	1	3	2	1	4
2	1	3	4	2	1	4
2	1	3	4	1	1	4
2	1	1	3	1	1	4
1	1	1	3	1	2	4
2	1	1	3	1	1	4
6	2	99	99	99	1	1
3	1	1	2	2	1	4
2	1	3	4	1	1	4
7	1	1	3	1	1	4
6	1	2	2	3	1	4
2	1	1	4	1	2	4
2	1	3	4	1	1	4
2	1	2	3	1	2	4
1	1	2	4	1	1	4
1	1	1	2	1	1	4
2	1	3	4	1	2	4
4	1	3	4	1	2	4
1	1	1	1	1	2	4
6	1	1	3	1	1	4
1	1	2	3	1	1	4
1	1	1	4	2	1	4
4	1	1	3	1	1	4
2	1	2	2	2	1	4
2	1	1	2	1	1	4
6	1	1	3	1	2	4
2	1	1	4	1	1	4
3	1	2	3	1	1	4
3	1	2	2	2	2	3
6	1	1	3	1	1	4
3	1	1	1	2	1	4
3	1	3	4	1	1	4
2	1	1	3	1	1	4
1	1	3	1	1	1	4
2	2	99	99	99	1	2

6	1	2	4	1	1	1
2	1	1	4	1	1	4
1	1	3	2	2	1	4
6	2	99	99	99	1	3
4	1	3	3	2	1	4
2	1	1	4	1	1	4
3	1	1	3	1	2	4
2	1	2	3	1	2	4
1	1	3	1	1	1	4
6	1	2	3	1	1	4
2	1	3	4	1	2	4
6	1	3	4	1	1	4
2	1	1	3	1	2	4
2	1	2	3	1	2	4
6	1	2	3	1	1	4
6	1	2	2	1	1	4
3	1	1	1	1	1	3
2	1	1	3	1	2	4
6	2	99	99	99	1	3
2	1	1	5	1	1	4
5	1	2	3	1	1	4
1	1	1	4	1	1	4
1	1	2	2	1	1	4
4	1	1	1	1	1	2
2	1	1	2	2	1	4
3	1	2	4	1	1	4
2	1	2	4	1	1	4
2	1	2	1	1	1	2
6	1	1	3	2	1	1
3	1	3	3	1	1	4
3	1	1	1	1	1	4
3	1	1	1	1	1	1
3	1	2	2	1	1	4
3	1	1	3	2	1	1
6	1	2	4	1	1	4
1	1	1	3	2	2	4
3	1	2	3	1	2	4
4	1	2	3	2	1	4
6	1	3	4	1	1	4
2	1	3	5	1	1	4
3	2	99	99	99	1	2
3	1	1	3	1	2	4
2	1	1	3	1	1	4
3	1	2	4	1	1	4
3	1	1	3	1	1	4
3	1	3	5	1	1	4
2	1	1	3	1	1	4

2	1	2	1	2	1	2
2	1	1	4	1	1	4
4	1	2	4	1	1	4
3	1	1	5	1	1	4
2	1	1	2	1	1	4
2	1	2	3	1	1	4
2	1	3	1	1	2	4
3	1	2	4	2	1	4
3	1	3	3	2	1	4
6	1	2	4	2	1	4
6	1	1	4	2	1	4
6	1	1	5	1	1	1
2	1	2	5	1	1	4
3	1	1	2	1	1	4
2	1	1	1	1	1	2
3	1	2	4	2	1	2
2	1	1	4	2	1	4
2	1	1	5	1	1	1
6	1	1	1	1	1	4
3	1	1	5	1	1	1
3	1	2	4	2	1	4
6	1	1	2	1	1	4
6	1	1	1	2	1	4
6	1	1	4	1	1	1
2	1	1	5	1	1	1
3	1	1	4	1	1	4
3	1	1	4	1	1	1
2	1	1	5	1	1	1
1	1	2	1	1	1	1
2	1	1	4	1	1	1
3	1	1	4	1	1	3
6	1	1	5	1	1	3
2	1	3	2	1	1	4
1	1	1	3	2	1	4
2	1	3	4	1	1	4
2	1	2	3	3	1	4
4	1	2	3	1	1	4
7	1	3	2	1	1	4
7	1	3	3	2	1	4
1	1	2	3	1	1	4
2	2	99	99	99	1	2
1	1	2	4	2	1	1
2	2	99	99	99	1	4
7	1	1	3	2	1	4
2	1	3	4	2	1	4
2	1	3	4	1	1	4
2	1	1	3	1	1	4

1	1	1	3	1	2	4
2	1	1	3	1	1	4
6	2	99	99	99	1	1
3	1	1	2	2	1	4
2	1	3	4	1	1	4
7	1	1	3	1	1	4
6	1	2	2	3	1	4
2	1	1	4	1	2	4
2	1	3	4	1	1	4
2	1	2	3	1	2	4
1	1	2	4	1	1	4
1	1	1	2	1	1	4
2	1	3	4	1	2	4
4	1	3	4	1	2	4
1	1	1	1	1	2	4
6	1	1	3	1	1	4
1	1	2	3	1	1	4
1	1	1	4	2	1	4
4	1	1	3	1	1	4
2	1	2	2	2	1	4
2	1	1	2	1	1	4
6	1	1	3	1	2	4
2	1	1	4	1	1	4
3	1	2	3	1	1	4
3	1	2	2	2	2	3
6	1	1	3	1	1	4
3	1	1	1	2	1	4
3	1	3	4	1	1	4
2	1	1	3	1	1	4
1	1	3	1	1	1	4
2	2	99	99	99	1	2
6	1	2	4	1	1	1
2	1	1	4	1	1	4
1	1	3	2	2	1	4
6	2	99	99	99	1	3
4	1	3	3	2	1	4
2	1	1	4	1	1	4
3	1	1	3	1	2	4
2	1	2	3	1	2	4
1	1	3	1	1	1	4
6	1	2	3	1	1	4
2	1	3	4	1	2	4
6	1	3	4	1	1	4
2	1	1	3	1	2	4
2	1	2	3	1	2	4
6	1	2	3	1	1	4
6	1	2	2	1	1	4

3	1	1	1	1	1	3
2	1	1	3	1	2	4
6	2	99	99	99	1	3
2	1	1	5	1	1	4
5	1	2	3	1	1	4
1	1	1	4	1	1	4
1	1	2	2	1	1	4
4	1	1	1	1	1	2
2	1	1	2	2	1	4
3	1	2	4	1	1	4
2	1	2	4	1	1	4
2	1	2	1	1	1	2
6	1	1	3	2	1	1
3	1	3	3	1	1	4
3	1	1	1	1	1	4
3	1	1	1	1	1	1
3	1	2	2	1	1	4
3	1	1	3	2	1	1
6	1	2	4	1	1	4
1	1	1	3	2	2	4
3	1	2	3	1	2	4
4	1	2	3	2	1	4
6	1	3	4	1	1	4
2	1	3	5	1	1	4
3	2	99	99	99	1	2
3	1	1	3	1	2	4
2	1	1	3	1	1	4
3	1	2	4	1	1	4
3	1	1	3	1	1	4
3	1	3	5	1	1	4
2	1	1	3	1	1	4
2	1	2	1	2	1	2
2	1	1	4	1	1	4
4	1	2	4	1	1	4
3	1	1	5	1	1	4
2	1	1	2	1	1	4
2	1	2	3	1	1	4
2	1	3	1	1	2	4
3	1	2	4	2	1	4
3	1	3	3	2	1	4
6	1	2	4	2	1	4
6	1	1	4	2	1	4
6	1	1	5	1	1	1
2	1	2	5	1	1	4
3	1	1	2	1	1	4
2	1	1	1	1	1	2
3	1	2	4	2	1	2

2	1	1	4	2	1	4
2	1	1	5	1	1	1
6	1	1	1	1	1	4
3	1	1	5	1	1	1
3	1	2	4	2	1	4
6	1	1	2	1	1	4
6	1	1	1	2	1	4
6	1	1	4	1	1	1
2	1	1	5	1	1	1
3	1	1	4	1	1	4
3	1	1	4	1	1	1
2	1	1	5	1	1	1
1	1	2	1	1	1	1
2	1	1	4	1	1	1
3	1	1	4	1	1	3
6	1	1	5	1	1	3
2	1	3	2	1	1	4
1	1	1	3	2	1	4
2	1	3	4	1	1	4
2	1	2	3	3	1	4
4	1	2	3	1	1	4
7	1	3	2	1	1	4
7	1	3	3	2	1	4
1	1	2	3	1	1	4
2	2	99	99	99	1	2
1	1	2	4	2	1	1
2	2	99	99	99	1	4
7	1	1	3	2	1	4
2	1	3	4	2	1	4
2	1	3	4	1	1	4
2	1	1	3	1	1	4
1	1	1	3	1	2	4
2	1	1	3	1	1	4
6	2	99	99	99	1	1
3	1	1	2	2	1	4
2	1	3	4	1	1	4
7	1	1	3	1	1	4
6	1	2	2	3	1	4
2	1	1	4	1	2	4
2	1	3	4	1	1	4
2	1	2	3	1	2	4
1	1	2	4	1	1	4
1	1	1	2	1	1	4
2	1	3	4	1	2	4
4	1	3	4	1	2	4
1	1	1	1	1	2	4
6	1	1	3	1	1	4

1	1	2	3	1	1	4
1	1	1	4	2	1	4
4	1	1	3	1	1	4
2	1	2	2	2	1	4
2	1	1	2	1	1	4
6	1	1	3	1	2	4
2	1	1	4	1	1	4
3	1	2	3	1	1	4
3	1	2	2	2	2	3
6	1	1	3	1	1	4
3	1	1	1	2	1	4
3	1	3	4	1	1	4
2	1	1	3	1	1	4
1	1	3	1	1	1	4
2	2	99	99	99	1	2
6	1	2	4	1	1	1
2	1	1	4	1	1	4
1	1	3	2	2	1	4
6	2	99	99	99	1	3
4	1	3	3	2	1	4
2	1	1	4	1	1	4
3	1	1	3	1	2	4
2	1	2	3	1	2	4
1	1	3	1	1	1	4
6	1	2	3	1	1	4
2	1	3	4	1	2	4
6	1	3	4	1	1	4
2	1	1	3	1	2	4
2	1	2	3	1	2	4
6	1	2	3	1	1	4
6	1	2	2	1	1	4
3	1	1	1	1	1	3
2	1	1	3	1	2	4
6	2	99	99	99	1	3
2	1	1	5	1	1	4
5	1	2	3	1	1	4
1	1	1	4	1	1	4
1	1	2	2	1	1	4
4	1	1	1	1	1	2
2	1	1	2	2	1	4
3	1	2	4	1	1	4
2	1	2	4	1	1	4
2	1	2	1	1	1	2
6	1	1	3	2	1	1
3	1	3	3	1	1	4
3	1	1	1	1	1	4
3	1	1	1	1	1	1

3	1	2	2	1	1	4
3	1	1	3	2	1	1
6	1	2	4	1	1	4
1	1	1	3	2	2	4
3	1	2	3	1	2	4
4	1	2	3	2	1	4
6	1	3	4	1	1	4
2	1	3	5	1	1	4
3	2	99	99	99	1	2
3	1	1	3	1	2	4
2	1	1	3	1	1	4
3	1	2	4	1	1	4
3	1	1	3	1	1	4
3	1	3	5	1	1	4
2	1	1	3	1	1	4
2	1	2	1	2	1	2
2	1	1	4	1	1	4
4	1	2	4	1	1	4
3	1	1	5	1	1	4
2	1	1	2	1	1	4
2	1	2	3	1	1	4
2	1	3	1	1	2	4
3	1	2	4	2	1	4
3	1	3	3	2	1	4
6	1	2	4	2	1	4
6	1	1	4	2	1	4
6	1	1	5	1	1	1
2	1	2	5	1	1	4
3	1	1	2	1	1	4
2	1	1	1	1	1	2
3	1	2	4	2	1	2
2	1	1	4	2	1	4
2	1	1	5	1	1	1
6	1	1	1	1	1	4
3	1	1	5	1	1	1
3	1	2	4	2	1	4
6	1	1	2	1	1	4
6	1	1	1	2	1	4
6	1	1	4	1	1	1
2	1	1	5	1	1	1
3	1	1	4	1	1	4
3	1	1	4	1	1	1
2	1	1	5	1	1	1
1	1	2	1	1	1	1
2	1	1	4	1	1	1
3	1	1	4	1	1	3
6	1	1	5	1	1	3

2	1	3	2	1	1	4
1	1	1	3	2	1	4
2	1	3	4	1	1	4
2	1	2	3	3	1	4
4	1	2	3	1	1	4
7	1	3	2	1	1	4
