# Supplementary material for: Health seeking behaviour and knowledge on neonatal danger signs among neonatal caregivers in Upper Denkyira East Municipality, Ghana
Source: BMC Pediatr. 2024 Jan 8;24:27. doi: 10.1186/s12887-023-04430-2 (PMC10773034; doi:10.1186/s12887-023-04430-2)
Supplement: Supplementary file 2 — Supplementary Material 2 [file 12887_2023_4430_MOESM2_ESM.pdf]

STUDY TOOLS (QUESTIONNAIRE)

We are conducting a study on health seeking behaviour and knowledge neonatal danger signs among caregivers in Upper Denkyira East Municipality, Ghana. Please be informed that the information you would give would be used for academic purposes only and would be treated with utmost confidentiality. You are therefore guaranteed complete anonymity and no identification of information is requested or will be transmitted with your completed questionnaire. Participation is voluntary.

Thank you for your co-operation.

SECTION A: Socio-demographic characteristic

1. What is your age……………………………………………
2. What is your marital Status: Single [ ] Married [ ] Co-habiting [ ] Divorced [ ] Separated [ ]
3. What is your religion: Moslem [ ] Christian [ ] Traditionalist [ ] Other (specify)…………
4. What is your tribe: Akan [ ] Ewe [ ] Ga-Adangbe [ ] Other (specify)…………
5. Your educational level: None [ ] Primary [ ] JSS [ ] SSS [ ] Tertiary [ ]
6. Occupation: Trader [ ] Artisan [ ] Farmer [ ] Health worker [ ] 5. Unemployed [ ] Other (specify)……….
7. Place of delivery: Hospital [ ] Home [ ] Other (specify)……

Section B: Knowledge on neonatal danger signs

8. Have you heard about neonatal danger signs before? (Tick as appropriate)

   Yes [ ] No [ ]

9. If yes, where did you hear it from?

   Health workers [ ] Friends [ ] Media [ ] Other (specify)…………..

10. What are neonatal danger signs?

    ……………………………………………………………………………………………

    ……………………………………………………………………………………………

    ……………………………………………………………………………………………

    Don't know [ ]

11. How will you recognize neonatal danger signs?

    When baby looks unhealthy [ ]

    When baby is crying [ ]

    When baby has fever [ ]

    Baby has diarrhoea [ ]

12. What do you do when your baby has danger signs?

    Go to the hospital [ ]

    Give paracetamol [ ]

13. Which conditions will make you send your neonate immediately to the health facility?

    Seizures [ ]

    Diarrhoea/vomiting [ ]

    Crying [ ]

    Not feeding well [ ]

Looking weak [ ]

Fever [ ]

Discharging cord [ ]

SECTION C: Health seeking behaviour

14. Where do you seek for health when your baby is sick?

Hospital [ ]

Chemical shop [ ]

Others, specify……………………….

15. If hospital, where do you receive care?

Public hospital [ ]

Mission hospital [ ]

Private hospital [ ]

16. How many times have you visited a hospital with this baby

Once [ ]

Twice [ ]

Thrice [ ]

Four [ ]

17. What do you do before taking your baby to the hospital?

Give paracetamol [ ]

Give ORS [ ]

Bath baby with cold water [ ]

Other (specify)……

18. Who prescribed the treatment above?

Self [ ]

Husband [ ]

Friends [ ]
